# New Interfaces and Approaches to Machine Learning When Classifying Gestures within Music

**DOI:** 10.3390/e22121384

**Published:** 2020-12-07

**Authors:** Chris Rhodes, Richard Allmendinger, Ricardo Climent

**Affiliations:** 1 NOVARS Research Centre, University of Manchester, Manchester M13 9PL, UK; ricardo.climent@manchester.ac.uk; 2 Alliance Manchester Business School, University of Manchester, Manchester M15 6PB, UK; richard.allmendinger@manchester.ac.uk

**Keywords:** interactive machine learning, Wekinator, Myo, HCI, performance gestures, interactive music, gestural interfaces, gesture representation, optimisation, music composition

## Abstract

Interactive music uses wearable sensors (i.e., gestural interfaces—GIs) and biometric datasets to reinvent traditional human–computer interaction and enhance music composition. In recent years, machine learning (ML) has been important for the artform. This is because ML helps process complex biometric datasets from GIs when predicting musical actions (termed performance gestures). ML allows musicians to create novel interactions with digital media. Wekinator is a popular ML software amongst artists, allowing users to train models through demonstration. It is built on the Waikato Environment for Knowledge Analysis (WEKA) framework, which is used to build supervised predictive models. Previous research has used biometric data from GIs to train specific ML models. However, previous research does not inform optimum ML model choice, within music, or compare model performance. Wekinator offers several ML models. Thus, we used Wekinator and the Myo armband GI and study three performance gestures for piano practice to solve this problem. Using these, we trained all models in Wekinator and investigated their accuracy, how gesture representation affects model accuracy and if optimisation can arise. Results show that neural networks are the strongest continuous classifiers, mapping behaviour differs amongst continuous models, optimisation can occur and gesture representation disparately affects model mapping behaviour; impacting music practice.

## 1. Introduction

The process of music composition can be considered interactive when using gestural interfaces (GIs) to create novel instruments [1] (or build upon existing ones [2]) to necessitate musical creativity. A GI is a device that measures bodily behaviours (gestures) to manipulate digital media. GIs capture biometric data, such as inertial measurement units (IMUs). When using GIs, new methods of human–computer interaction (HCI) can be realised within music composition.

Novel GIs have always induced creativity within interactive music history. This is evident when observing the history of GIs within music, spanning from the theremin (1917) [3] to the analogue synthesizer (1970s) [4], Michel Waisvisz’s GI ‘The Hands’ in 1984 [5] (a mobile GI allowing for musical embodiment), which was produced one year after the establishment of the Musical Instrument Digital Interface (MIDI) protocol, then Jon Rose’s newly created digital/MIDI violin bow (namely the K-bow, 1987) [6] and, lastly, Atau Tanaka et al.’s 1993 artistic group *Sensorband* [7], who took inspiration from Waisvisz’s earlier work, investigating how a range of wearable sensors affected the digital signal processing (DSP) of live sound/music. In recent times, brain–computer interfaces (BCIs) using electroencephalography have been used to broaden our understanding regarding the embodiment of music composition [8]. However, BCIs are not ideal for music composition because they utilise numerous electrodes fixed atop the head. As a result, their mobility is limited. In 2015, the release of the Myo armband [9] provided an opportunity to use a mobile interface for better gestural control within interactive music practice. The Myo facilitated access to two types of biometric data: IMU and electromyographic (EMG). Although IMU data offered by the Myo (and similar interfaces) are not novel, EMG data are very useful as they capture skeletal muscle activity, granting users the ability to map muscular behaviour to digital media.

Past research using the Myo GI has used conditional statements to process biometric information [10]. Although conditional statements can be used with interface data and music composition, they are inefficient. This is because misclassification of musical gestures can occur if a machine learning (ML) approach is not taken [10]. Using ML would therefore provide better accuracy when stimulating music systems. Wekinator, an interactive ML software, makes this possible [11]. Wekinator is built on the WEKA (https://www.cs.waikato.ac.nz/ml/weka/) framework. It allows real-time data to be used as a model input (from GIs) and contains a simple graphical user interface (GUI) when building ML models. Previous literature has used Wekinator to classify instrument articulations [12], map colour from sound within virtual reality [13] and duet with a GI using LEDs [14]. Moreover, there is a scarcity of literature investigating performance gesture classification in Wekinator with a focused quantitative method; only a demonstrative method using qualitative evaluations [12]. Nevertheless, a small number of studies exist that feature quantitative aims, but without such aims being the focus of those studies. Studies using Wekinator have also investigated how a hybrid of subjective and quantitative evaluation [15] can be used with gestural datasets (besides EMG data) in music practice.

Previous research has seldom explored model evaluation when using EMG data to train ML models within Wekinator, although ML practice with rich gestural datasets in music is of high value; literature in this field remarks, ‘… there is a need to create evaluation models to assess their suitability for real world music performance situations’ [16]. In addition, literature in this topic rarely explores the use of EMG data to classify highly nuanced physical actions in music (e.g., the classification of each individual digit of the hands during piano performance), despite being able to do so, if current post-processing (i.e., feature extraction) methods are used with GIs. Importantly, such literature does not also assess the impact of model optimisation and if suggestions can be made when pairing gesture type to model choice. Areas such as the number of model examples used, comparing performance gesture representation during training, or how historic data can be utilised to make gestural predictions in music, are not addressed. Having more insights into these topics would potentially inform us about which gestures can be predicted, and the volume of data required to achieve high prediction accuracy.

Moreover, how we capture musical actions is an area of importance. Performance gesture representation is important to study, prior to model training, because it can disparately affect artistic outcomes. Literature in this field has investigated two types of performance gestures within ML practice, prior to model training, when using EMG data and GIs (i.e., the Myo): static and dynamic [17]. However, this is without objective evaluation. Therefore, the fact that we have a choice in how we represent a gesture in music before model training is a problem because we do not know how they will be represented by an algorithm—or any resulting artistic consequences. Our approach to gesture representation is discussed further in Section 3.2.1.

Therefore, we provide a solution to the problem that ML models can be processed and created differently within Wekinator when using performance gestures and the Myo GI. In addition, we address that EMG data can be used to create unique gestures for music system interaction, in lieu of predefined gestures (via the Myo Software Development Kit (SDK)) within music research [10,18]. We investigate which ML models are the most reliable when predicting three particular music actions (for piano) within interactive music, and how model accuracy can be improved under specific conditions. If successful, this research enquiry will better inform the artistic community regarding the Myo GI (and similar interfaces) when selecting an efficient ML model within Wekinator to predict our studied performance gestures. We investigated these points by using current post-processing methods with gestural EMG/IMU data, optimising models, assessing model example lengths on accuracy, using historic gesture data to make predictions and exploring how gesture representation choice affects model prediction behaviour and accuracy.

Our results show that specific ML models are most efficient when selected based on performance gesture representation and biometric data type. The results also show that model choice provides disparate music outputs via differences in model signal behaviour and post-processing EMG data models generally improves model accuracy, in comparison to such models which do not use post-processing. We also see that model parameter optimisation can improve model accuracy, and using historic data to run trained models provides very similar behaviours as when real-time data are used. Lastly, we also observe model example lengths can affect model accuracy. The next section of this paper outlines and critically assesses literature in the field of gesture recognition with ML and music. Section 3 then discusses the GI that we used for this study (the Myo armband), our planned performance gestures to evaluate ML practice, our approaches to gestural representation, data acquisition, data processing, all ML models offered by Wekinator, the use of historic gesture data, example length and model optimisation. This is followed by the study results (Section 4), a discussion of the application of the three studied gestures within interactive music practice (Section 5) and conclusions (Section 6).

## 2. Literature Review

Studies attempting to inform the user of which ML model is most apt to use when classifying gestures in music, using a popular ML software such as Wekinator, and rich gestural datasets (i.e., EMG data) are sparse; see Table 1 for a summary of key gesture recognition studies, in music, elucidating this. Within the field of gesture classification, several studies have previously used optical datasets and ML, using peripherals such as the Leap Motion and Kinect [19]. Other studies have used images and videos to classify gestures [20]. Other studies in this area have compared ML algorithms using the Kinect/optical peripherals [21] in order to find the most efficient model. Optical peripherals have also recently been used in virtual reality, utilising ML for gesture prediction, in order to track user hands [22]. However, optical datasets can be inefficient for gesture classification because their efficacy may be affected by environmental conditions (i.e., differences in distance between gesture and camera, fluctuations in scene lighting and occlusion) compared to other gestural datasets, such as EMG [23].

Literature within the scientific community has been integral when informing how we should use complex EMG information from available GIs. In particular, Arief et al. [29] showed that the mean absolute value (MAV) feature extraction method is one of the most efficient when working with EMG data. The MAV is an integral of EMG data and estimates muscle amplitude via EMG signals [29,30]; it is represented as follows, where $X_{k}$ denotes kth model attribute input and *N* denotes attribute input sample size:

(1) $$MAV=\frac{1}{N}\sum_{k=1}^{N}\left|X_{k}\right|$$

Research within prosthetic and rehabilitative studies have used gestural devices, measuring EMG data, to control peripherals. A study by Phinyomark et al. [31] found that gestures could be classified (via a support vector machine model) through the use of the Myo armband and EMG data. Classification was done by using the MAV as one of the feature extraction methods, based on the findings by Arief et al. [29]. Interestingly, the study found that EMG information could be used to classify gestures with >72% accuracy.

Studies using EMG data to classify gestures within music have investigated how gestures can be used to augment instrumental practice. A study by Ruan et al. [25] looked at how an ‘Air-Ukulele’ could be created via the use of ML to classify EMG information. The classification process used the MAV function informed by the findings made by Arief et al. [29]. In this work, the authors used a multilayer perceptron neural network (MPNN) to classify the thumb, index and middle fingers. The authors noted a high accuracy score for the study, where they achieved a gesture classification accuracy of 80.8%. The authors framed their experimental method by investigating which conditions the MPNN performed most accurately. The authors also cited a 90% model training accuracy (TA); i.e., a 30% TA improvement through optimisation.

In the last decade, there has been a seismic development in how music practitioners use peripherals to develop their practice. This has been in part because of work done by Fiebrink [11] and the development of an interactive machine learning (IML) system—Wekinator. The success behind Wekinator, within artistic communities, is because of how it bridges complex logic (i.e., ML theory and algorithms) with an improved accessibility of ML via an intuitive GUI, whereby the GUI allows users to record examples, from any kind of network peripheral, into a specific ML model; the user can choose between classification, continuous (i.e., regression) or dynamic time warping (DTW) models (more on this in Section 3.4). IML differs from conventional ML practice for this reason; it connects an informed understanding of ML implementation, programming languages and the real world application of ML. Even though IML has improved the accessibility of applied ML within the artistic community, there is still a lack of understanding as to which ML models are the best to use within a particular artistic context. In this sense, there is not yet an informed framework—only experimental application.

Recent studies using Wekinator to investigate how IML can be used to drive sophisticated interactive gestural systems, in music, have seldom used several ML models to do so [17,26]; instead, they focus on the usability of a few models to drive such a system. This means that an ML model is used to achieve a particular research aim but optimal model choice is uninformed. A 2017 study by Dalmazzo and Ramirez [24] classified four fingers of the left hand (i.e., index to ring fingers) to build an ’Air Violin’ via the use of two ML models: a decision tree J48 (DT) and a hidden Markovian model (HMM). This study is particularly interesting because the authors compared which model was better suitable for gesture classification. They concluded that both models were highly accurate for their focus; citing a 91.4% accuracy for the highest DT finger classification accuracy and 95.1% for the HMM. Most interestingly, the authors state that the DT model (in Wekinator) can be optimised through fine-tuning the parameters of the algorithm.

Fiebrink [15] initially explored how gestural datasets (not using EMG data) in music practice can be investigated through a blend of subjective and quantitative evaluation. In this study, Wekinator users evaluated models via direct (human) evaluation and also objective evaluation, then subjectively modified model parameters until they were satisfied with how the model was performing; however, a specific model was not defined for use when classifying specific music gestures and model choice, or optimisation, was not informed prior to the decision process. Interestingly, although the study did attempt to evaluate how music practitioners could benefit from using IML with performance gestures, a methodology for gesture representation within the dataspace was not investigated. However, gesture representation is challenging within IML practice because gesture representation methods are not clearly defined. This is because there is no strict taxonomy between best IML and gesture performance practice. However, noted studies have influenced gesture representation within contemporary IML focuses, using the recognised mapping by demonstration principle [32] to arbitrarily allow a user to define gesture representation by listening to sound examples; in this sense, the user maps their gestural perception of predefined sound to stimulate music.

Modern literature in the field of IML has built upon gesture representation research. However, such literature uses the mapping by demonstration principle [32] to provide a focused approach to gesture representation within IML practice. A study by Tanaka et al. [17] narrowed the research focus by asking workshop participants to use two ML methods to represent participant-designed gestures, when using EMG data and GIs (i.e., the Myo); these ML methods were static and dynamic. In the study by Tanaka et al. [17], participants were given a choice of how they wanted to represent a gesture they had designed via training specific ML algorithms; one static method and three dynamic methods. Gesture representation was defined via four different types of ML approaches; three were based on using a neural network (NN), within Wekinator and one based on a hierarchical hidden Markov model (HHMM) for music generation. The ML methods were chosen because of the different artistic affordances they provided to the user when defining a gesture of their choice. The qualitative results from the study [17] suggested that gesture representation model choice is wholly based on subjectivity. Therefore, the fact that we have a choice in how we represent a gesture in music before model training is a problem. This is because we do not empirically understand how they will be represented by an algorithm through the gesture choice and artistic consequences that follow. Thus, the study investigated the role that model type has in aiding artistic decision. However, the study did not evaluate whether gesture type affects model accuracy, or algorithmic behaviour, when applied to acoustic instruments or digital musical instruments (DMIs) (DMIs are “... devices in which gestural control and sound production are physically decoupled but digitally connected according to a mapping strategy.” [33]) practice. Our approach to gesture representation is discussed in Section 3.2.1.

Other recent studies have further investigated the role that gesture representation has in sound-action mapping when using IML to build DMIs and sound-producing ’air’ guitars. A study by Erdem et al. [27] investigating the development of an air guitar DMI (via ML methods) focused on *excitation* (Excitation is a phase where there is energy transfer between a music performer and a musical object. The excitation phase is preceded by a prefix (movement trajectory towards the point of contact with the musical object) and followed by a suffix (movement away from the musical object) [34]) actions when implementing sound-action mapping for the air guitar. The authors used several excitation categories to investigate whether action-sound gesture coupling can be used to create action-sound mapping for a novel DMI (air guitar) which does not rely on a physical controller (i.e., a physical instrument), using EMG data from the Myo GI to do so. The study used a recurrent neural network (RNN) model and trained it with data from semi-professional musicians/participants to predict guitar gestures. The authors then created an RNN model by mapping EMG data to the root mean square (RMS) of the instrument’s audio signal and producing a synthesised guitar sound (i.e., a physical model). The results from the study did not include an evaluation of methods used. Ultimately, the study illustrated how a focused approach to gesture representation and IML is necessary when building a novel DMI.

Due to the fact that IML is in its infancy, it is currently unclear what the implications for model choices are. Schedel and Fiebrink’s [12] study on cello bow gesture recognition highlights this importance when they noted how optimisation was shown to be a factor when the performer, in the study, tried to improve model accuracy in Wekinator. Similarly, preliminary investigations by Ruan et al. [25] and Dalmazzo and Ramirez [24] show that model optimisation can be beneficial when improving prediction accuracies for use in music. In terms of the impact of example length on model behaviour, the earlier noted study by Ruan et al. [25] mentioned that their MPNN classification model could improve if more training data were introduced.

Vast literature within the field of IML looks at the real-time application of supervised learning models. The use of historical data to make predictions, however, is rarely addressed despite having offline advantages. Using historic data is no novel concept within wider ML practice [35,36,37]. Using historic data can benefit IML practice in music because capturing biometric datasets from virtuosic musicians, for example, can be used to better inform and optimise ML models. This would be especially useful when addressing the higher-level goals of IML and music research. A study by Dalmazzo and Ramírez [28] used a HHMM to train a system to automatically detect violin bowing gestures. The system was capable of predicting such musical actions with over 94% accuracy. However, the aim of the study was to provide real-time training to violin students; in this sense, the application of this study was pedagogically oriented.

## 3. Methodology

**Figure 1:**
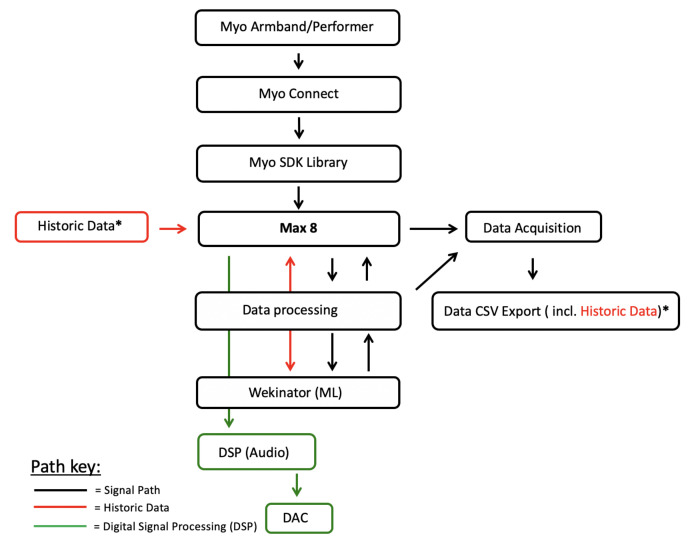
Illustrative project pipeline of biometric data acquisition from the Myo gestural interface (GI), data processing and machine learning (ML). The end of the pipeline displays digital signal processing (DSP) and digital to analogue conversion (DAC) when creating musical output.

Our methodology follows the project pipeline in Figure 1. We use this pipeline to investigate how three specific performance gestures, for piano practice, can be acquired from a GI (Myo armband), processed, communicated to different ML models (in Wekinator) and used to create a sonic output. Section 3.1 details the GI that we used to conduct our study, the data it transmits and issues surrounding the use of the interface. Section 3.2 outlines three performance gestures we designed to test ML model behaviour across all models (classifiers, continuous and DTW). This section also outlines our investigation, observing if gesture representation choice affects model behaviour and accuracy. Section 3.3 then discusses data acquisition from the Myo GI used in this study and our approach to structuring and processing the data. Lastly, Section 3.4 explains the ML approaches we apply to retrieved biometric data, using real-time and historic data to do so, as well as our method behind optimising models and observing if model example length affects model accuracy/behaviour.

### 3.1. Gestural Interfaces and Biometric Data

GIs give us access to raw biometric data when building ML models with performance gestures. We used the Myo armband GI for this study.

#### 3.1.1. Gestural Interface: The Myo Armband

The Myo armband GI provides access to raw IMUs and 8-channels of 8-bit EMG data. The Myo transmits data via Bluetooth and communicates EMG data at 200 Hz and IMU data at 50 Hz [10]. The armband can be adjusted to the forearm circumference between 19 and 34 cm and weighs 93 g [28]. The Myo interface streams data to a USB Bluetooth receiver within a distance of <15 m [38]. Data are retrieved from the Myo SDK developed by Thalmic Labs [18]. The Myo is a unique GI because it offers access to raw EMG data; such an interface is difficult to obtain at consumer level.

#### 3.1.2. Transmitted Data

**Figure 2:**
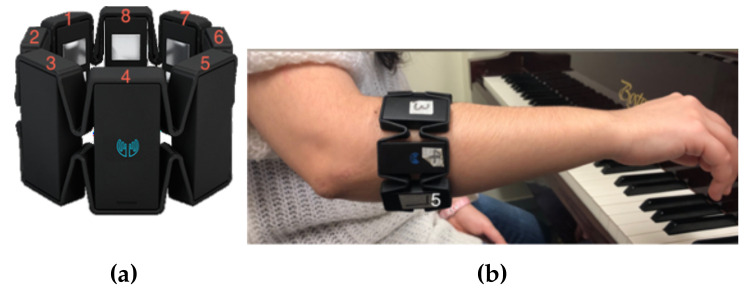
(**a**) The Myo GI, where all electromyographic (EMG) electrodes (8 channels) are shown. (**b**) Standardised placement of the Myo GI when acquiring biometric information (inertial measurement units (IMUs) and EMG).

The Myo provides two types of biometric data: IMU and EMG data. IMU data streamed from the Myo are communicated via three orientation parameters: acceleration, gyroscope and quaternions. Acceleration and gyroscope data parameters use 3 axes (X, Y, Z) to quantify orientation, whereas quaternions use 4 (X, Y, Z, W). Quaternion units can be used to calculate Euler angles yaw, pitch and roll (we used Myo Mapper (https://www.balandinodidonato.com/myomapper/) external software to do this). EMG arm activity is measured from the Myo across 8 separate electrodes, as seen in Figure 2a. We used only EMG and pitch data within this study. EMG data is returned with a range between −1 to 1 and pitch data is returned (from Myo Mapper) with a range between 0 to 1. We post-processed these returned values as explained in Section 3.3.1.

#### 3.1.3. Usage Issues of the Myo

Transmitted data from the Myo are prone to data validity issues. Namely, calibration and the placement of the interface.

**Calibration**: Muscle activity within a Myo user’s arm is unique to the individual [39]. Therefore, users must calibrate the Myo to ensure data reliability. Failing to calibrate the Myo before use will invalidate acquired data as measurement is altered; for example, not providing a point of origin in space for IMU data.
**Placement**: Careful placement of the Myo must be observed and standardised. See Figure 2b. This is because incorrect placement will skew all EMG results. A change in rotation of the Myo, between future participants or users, will change the orientation of electrodes (measuring EMG data) and therefore affect data or system validity. We placed the Myo in the same position for data acquisition, model training and testing. This also allowed us to observe how gestural amplitude relates to the returned ML model accuracies.

### 3.2. Planning Performance Gestures for Model Testing

We developed three performance gestures (performed by the first author) for augmenting piano practice. Using the piano instrument would yield a primary focus for gesture prediction and interactive music composition. All gestures used in this study can be considered extended techniques because they use ML to stimulate additional instrument interactions; in other words, unconventional piano performance methods. We use one Myo worn on the right arm to make the process of understanding the results easier. We also used three different performance gestures (with different biometric datasets) when training differing ML models. This is because the ML models offered in Wekinator compute data in different ways; e.g., a performance gesture that navigates between two points in space on the *y* axis—points $y^{1}$ and $y^{2}$—can be classed as a static or continuous gesture. On a data level, this would be shown as floating-point values (continuous) or a single integer value (classifier). However, the two states merged to one value can be reviewed as a suitable input for a DTW model. This is because the DTW algorithm continuously predicts performance gestures per iteration and does not represent such gestures as floating point values or classified states. Thus, deciding which metric/model to use is problematic. The performance context within piano practice is therefore the key factor when pairing model choice to training data. We isolated the performance context by providing a very strict focus on three performance gestures, which are as follows:

1. **Right arm positioned above head (gesture 1)**: This gesture requires the performer to extend their right arm above the head, linearly. We used the Euler angle ’pitch’ (IMU) to measure this gesture, yielding low data variation because of the linearity of the dataset. Due to this linearity, the model input will position itself very closely to model output. This gesture is therefore most suited to a continuous model. This gesture does not use EMG data; it is used in this study to see if the same behaviour occurs between ML models trained with EMG and IMU data. Musically, it is aimed to measure the dynamic movement of pianist’s arms, typically performed theatrically during performance; if a simple raising of the arm demonstrates any significance amongst model accuracy/behaviour, we can then elaborate on better complex IMU-measured gestures. This gesture was inspired by piano practice, as seen in a piece of music by [40] called *Suspensions*, where the performer lifts the arm above the head to shape sounds over time via DSP; demonstrating how theatrical piano gestures (which do not traditionally produce sound) can become musical when applied to interactive music practice. The performed strength (amplitude) of this gesture is relative to returned model accuracies. We measure it via observing minimum and maximum values for Euler angle pitch and the data distribution of each trained model (see Section 3.3.1 for more information on this process). A video example of the performance of this gesture can be found at the following link: performance of gesture 1 (https://www.dropbox.com/s/a250u5ga3sgvyxu/Gesture1.mov?dl=0).
2. **Spread fingers and resting arm (gesture 2)**: This gesture is executed by: (a) extending all digits on the right hand (b) putting the arm back to a relaxed position. Gesture position (b) was developed to examine model prediction efficacy of position (a) when shifting between the two states. This gesture navigates between two fixed states. Therefore, it was logical that a classifier model was most suitable for this gesture. This gesture targets all 8 EMG electrodes on the Myo interface (see Figure 2b). This gesture is inspired by the need of a pianist to access a number of musical intervals (an interval is the distance in pitch between two different musical notes, measured by numerical value and determined musical quality [41]) with one hand; when playing melodies, chords, scales and arpeggios. In piano practice, the ability of the pianist to play large intervals is dependent on an individual pianist’s hand span; defined by age, gender and ethnic differences amongst pianists [42]. The average interval range available to pianists is from a second to a maximum of a ninth or tenth [42]; the latter being harder to achieve. This gesture negates such differences because the Myo GI measures EMG activity relative to the pianist and translates it in to a musical outcome; a large desired interval (i.e., a 10th) can therefore be digitally mapped during interactive music practice, if unable to be played. Through musical repertoire, this gesture was influenced by the tenth musical interval (i.e., found in the right hand part, in bar 3, of Harry Ruby’s *tenth interval rag* [43]), where the piece is composed around the difficult to perform (on average) tenth interval. We measured the performed strength of this gesture via the minimum and maximum MAV values returned across all 8 EMG electrodes of the Myo armband, as well as observing data distribution; we used the MAV to measure performance strength because it is typically used with EMG signals to estimate muscle amplitude [30] (refer to Section 3.3.1 for more information on this method). A video example of the performance of this gesture can be found via the following link: performance of gesture 2 (https://www.dropbox.com/s/mor7ccjyyhr13ry/Gesture2.mov?dl=0).
3. **Scalic fingering (gesture 3)**: This gesture is performed by extending and isolating pressure on each individual digit of the right hand, including: thumb (i); index finger (ii); middle finger (iii); ring finger (iv); little finger (v). This gesture was created to understand and execute a nuanced detailing of EMG data, as muscle behaviour for moving individual digits is much more finely detailed than gestures activated by large and pronounced muscle groups (i.e., gesture 2) [25]. It was also chosen because literature on this topic does not use all five digits when examining such a gesture, whereas the maximum number of digits used to train a ML system is four [24,44]. Musically, the use of all five digits is fundamentally important when playing piano music; through scales, chords, arpeggios and melodies. We base this gesture on the first 5 notes (with fingering pattern) found in the upper bass clef voicing, within the first bar of Hanon’s scale exercise (no.1 in C), taken from his iconic piano exercise book *The Virtuoso Pianist* [45]; this is because fingers i–v are used to achieve focused pitches/fingering. Albeit, even though this gesture focuses on a single scale, it should be noted that scalic playing compromises wider piano playing.
  This gesture aims to allow performers to create a piano DMI, through emulation of the Hanon scale. Finger ML classification can be used, via this gesture, to replicate piano performance away from the instrument; in other words, playing ’tabletop’ piano. The advantage of this approach is an increased mobility of piano performance; however, it also means that this approach could be further used to simulate piano performance and build novel DMIs (e.g., via interacting with 3D digital instruments). The mobile instrument approach has particular interest in the field for this reason [24,25,27]. The identification of fine EMG detailing is done, for this gesture, via post-processing and the application of a MAV function (discussed in Section 3.3.1). The DTW model has been targeted for this gesture because each individual digit needs to be accounted for as a single-fire, discrete, state. This gesture targets all 8 EMG electrodes on the Myo interface (see Figure 2a) and uses the MAV function, across all electrodes, to estimate the performance amplitude of this gesture (discussed in Section 3.3.1). A video example of the performance of this gesture can be found via the following link: performance of gesture 3 (https://www.dropbox.com/s/tg6wa7uap38y3c9/Gesture3.mov?dl=0).

#### 3.2.1. Onset Vs. Static Gestures for Model Training in Wekinator

**Figure 3:**
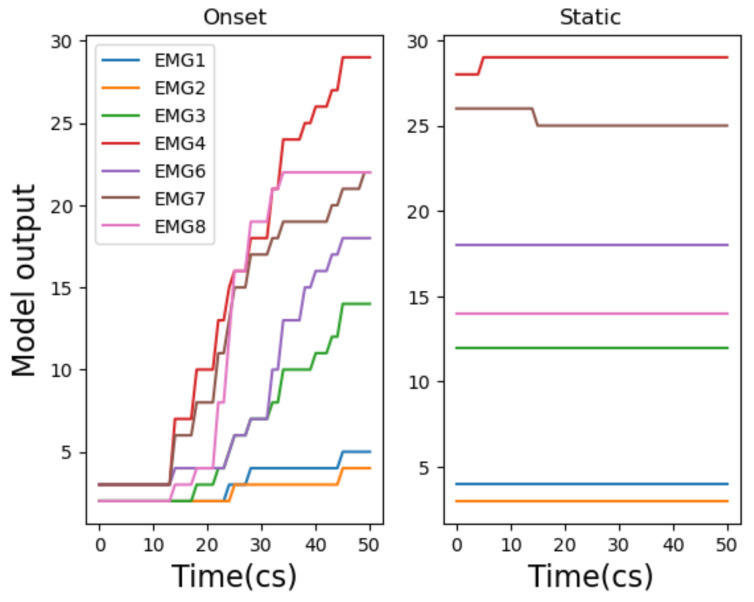
Onset model: the first 50 cs of gesture 3 (i) performed in an onset state, where gesture onset is included. Static model: the first 50 cs of gesture 3 (i) performed in a static state, where gesture onset is omitted. Cs = centiseconds.

Using Wekinator, we observed two approaches to model training via gestural input: a ‘static’ approach and ‘onset’ approach. A static model here is defined as a model that has been trained using gestural data that omits the onset of a gesture and instead uses the performance of the gesture in a static state (see right of Figure 3). This causes data variation to be low and—as a result—improves model accuracy. Perversely, an onset model includes the onset of a gesture, increasing data variation and therefore decreasing accuracy (see left of Figure 3). Comparing static and onset model approaches is important to consider in our approach to ML and music because, as a temporal art, timing via gesture is important.

### 3.3. Data Structuring and Acquisition

**Figure 4:**
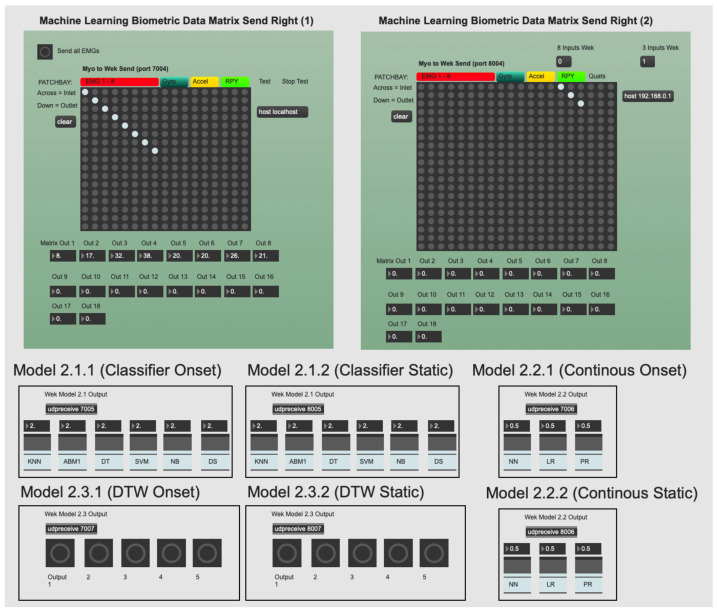
Software built by the first author (in Max 8) for routing biometric data parameters from the right Myo (done via the displayed matrices) and sending it to Wekinator; returned onset/static classification data from Wekinator can be seen below the matrices.

Data were structured from the Myo (placed on the right arm) through software developed by the first author within the Max 8 application (Max 8 is a GUI programming environment primarily used by artists and musicians. More information regarding Max 8 can be found here: www.cycling74.com). The developed software channelled the Myo application programming interface, taken from the Myo SDK, to Max 8 through the Myo for Max (https://www.julesfrancoise.com/myo) external. Two pieces of software were developed in Max 8: an environment to retrieve, process, send Myo data to Wekinator, receive Wekinator classifications and apply DSP (see Figure 4 for an example of this environment), and a second environment was built to record gesture performance against Wekinator classification behaviour (for offline analysis). Upon receipt of the Myo data within Max 8, data were pre-processed, post-processed and saved as a .csv file for analysis offline. The first author performed all gestures for data acquisition, model building and evaluation. We used one performer in order to observe model behaviours and derive conclusions over three specific performance gestures; in later work, we intend to invite more participants and begin to generalise such gestures amongst different music performers.

**Acquisition**: Data were acquired from the Myo SDK at 10 ms per entry. This acquisition frequency was deemed ample in order to see significant data trends. Gestures 1 and 2 were recorded with 4 iterations over 16 s. Gesture 3 was recorded with 5 iterations in 20 s. Gesture 3 was recorded with a longer length of time because the gesture is much more complex than gestures 1–2. We used a digital metronome to synchronise the data acquisition software with gesture performance.
**Pre-processing and structuring**: Data were pre-processed following acquisition via unpacking and structuring/labelling all Myo data (i.e., IMU and EMG) to an array (see Section 3.1.2 for a summary of data types).

#### 3.3.1. Data Post-Processing

Gesture 1 and gestures 2–3 use different datasets from the Myo. Therefore, both groups of gestures use different data processing methods. Gesture 1 uses pitch Euler angle from the Myo and gestures 2–3 use EMG data.

Where gestures 2–3 are concerned, the EMG data acquired were post-processed via scaling the data and applying a MAV function (the latter as discussed in Section 2). Each EMG parameter was taken after acquisition, an absolute (ABS) function was applied (reducing the polarity of the EMG signal) and the resulting range (0–1) was scaled between 0 and 100; this is expressed within Equation (2), where *x* is each raw EMG parameter (with minimum and maximum boundaries as 0 and 1 duly), *y* denotes the scaling variable (showing the minimum and maximum boundaries of said scaling function, which are 0 and 100, respectively) and $x^{\prime }$ is the scaled EMG parameter. As the minima of *y* and *x* are equal to 0, Equation (3) simplifies Equation (2).

(2) $$\begin{array}{c} x^{\prime }=y\min+\frac{\left(y\max-y\min)(|x|-|x|\min\right)}{|x|\max-|x|\min} \end{array}$$

(3) $$\begin{array}{c} x^{\prime }=100\left|x\right| \end{array}$$

The data were scaled for ease of use with other applications than ML model training, such as data visualisation and DSP mapping. After the ABS function was applied to each EMG parameter (in our case, done via the scaling process), the mean reading was then calculated. This process was done automatically following the scaling step. Equation (1) expresses this MAV function. This method of feature extraction, in particular, is known as MAV and has been shown to be the most effective method of feature extraction when working with time series EMG data provided by the Myo [29]. MAV is an amplitude estimator of muscle activity from EMG data [30]. Using the MAV is also useful because it stabilises and reduces the dimensionality of often complex and non-static EMG data [46]. We estimated the performance amplitude of gestures 2–3 via using the MAV across all 8 EMG electrodes of the Myo armband; done via reading minimum and maximum EMG MAV values to determine a range of applied strength and by presenting data distribution behaviour through boxplots (see Appendix C, Table A1, Table A2, Table A3, Table A4, Table A5 and Table A6 for such quantified EMG gestural amplitudes with all key model/gesture types used). This is important to note because performed gestural strength is relative to model accuracies presented in this study. Processed MAV EMG data were used to train models by first using an activation threshold function, acting as a window. This window is applied by calculating the mean of the last 10 readings of each Myo EMG parameter (i.e., electrodes 1–8) and using a threshold value for activation; sending the values for training in Wekinator. We use an arbitrary threshold of ≥2 from experimentation. Thus, the system can detect when a user is performing a gesture and resets all MAV calculations (across all EMGs/IMUs) when nothing is performed. This allows models to listen to focused streams of the datasets (i.e., only when gestures are being performed).

Gesture 1 uses orientation pitch data and not EMG. Therefore, we did not process gesture 1 data via MAV. Instead, we acquire pitch Euler angle data from the Myo via Myo Mapper and scale the returned dataset from range 0.0–1.0 to 0–100. We scale the returned data accordingly for clarity. This is expressed within Equation (4), where *x* is the Euler pitch parameter (where the seen minimum and maximum values are between 0 and 1 duly), *y* shows the scaling variable (whereby the minimum and maximum values have limits between 0 and 100 respectively) and $x^{\prime }$ denotes the scaled pitch output. As the minima of *y* and *x* are equal to 0, Equation (5) simplifies Equation (4).

(4) $$\begin{array}{c} x^{\prime }=y\min+\frac{\left(y\max-y\min)(x-x\min\right)}{x\max-x\min} \end{array}$$

(5) $$\begin{array}{c} x^{\prime }=100x \end{array}$$

It is important to note that the returned pitch dataset from Myo Mapper is bipolar (i.e., measuring movement in two different directions) and the user is required to ’set origin’ (i.e., calibrate) within the software before gestural performance. After doing this, the pitch is set to a value of 0.5 (a value of 50 following our described post-processing stage), thus ensuring movement can be detected both downwards and upwards from the point of origin. Lastly, we utilised the ’flip’ option within Myo Mapper, applied to the pitch dataset. This allows an upwards gestural movement to take a raw value closer to 1.0 rather than 0.0; this is also for clarity in the returned dataset. We measured the gestural amplitude of gesture 1 via observing minimum and maximum model input values when using pitch data, as well as observing data distribution (see Appendix C, Table A1, Table A2, Table A3, Table A4, Table A5 and Table A6 for quantified gestural amplitude values across all core model/gesture types used).

### 3.4. Machine Learning within Wekinator

Wekinator is an interactive ML software that was created by Rebecca Fiebrink in 2009 [11]. It features an accessible GUI and is built on the WEKA framework (using Java). Wekinator provides three ML model categories for input data: continuous models, classifiers and DTW. The next subsections explain these models in greater detail, followed by an overview of how these models can be trained and optimised.

#### 3.4.1. Ml Models in Wekinator

**Continuous models:** Continuous models in Wekinator include (i) linear regression (LR), (ii) polynomial regression (PR) and (iii) neural network (NN). These can be considered regression models. They are useful for modelling real-time model inputs versus model outputs over time. They do not make categorical classifications, unlike classifier or DTW models offered in Wekinator. They are useful in music practice because their model output type (regressive) can be used to continuously alter an audio signal (via DSP) over time. However, each continuous model in Wekinator provides different methods and behaviours to do this (see Appendix A for an explanation of how each continuous model functions and their application to music).
**Classifier models:** Classification models in Wekinator include (i) k-nearest neighbour (k-NN), (ii) AdaBoost.M1 (ABM1), (iii) DT, (iv) support vector machine (SVM), (v) naive Bayes (NB) and (vi) decision stump (DS). These models are important to understand because their gestural predictions affect interactive music practice. A weak classification model is not reliable or desirable to use within a temporal art form, relying on immediacy. Therefore, how they arrive at those decisions is of interest. Their output is categorical (e.g., classes 1–5) and they are useful in music practice for defining fixed sequence points within a gesture. However, they do not apply a real-time mapping to audio signals, unlike continuous models do. See Appendix B for details regarding how the classifier models offered in Wekinator operate at an algorithmic level and how they apply to music practice.
**Dynamic time warping:** DTW is a model that looks to predict a pattern over a time series. It is best used when attempting to predict a pattern, within an attribute, in spite of fluctuations in timing [47]. The algorithm aligns the trained model with fluctuations over the time series, hence the namesake. This model is very useful in music practice because it attempts to predict a music gesture (using the trained template) regardless of timing issues. Therefore, it compensates for subtle timing variances in how a music performer actions a specific gesture; a common issue in music practice. The DTW model output is a single-fire message every time the model predicts a gesture; useful in music when a performer wants to trigger a fixed music event only when a gesture is performed.

#### 3.4.2. Training Ml Models in Wekinator Using Real-Time Data

Following processing, training data were sent from Max 8 to each model input in Wekinator through Open Sound Control (OSC) and User Datagram Protocol (UDP). We used UDP to send OSC data packets to specific ports in Wekinator. All models were measured using performance gestures 1, 2 and 3 (detailed in Section 3.2). Each gesture was recorded at a frequency of 10 ms per example. Gestures 1–2 were performed over a period of 16 s and gesture 3 was performed over a period of 20 s; we designate 8 s per class for gestures 1–2 and 4 s per class for gesture 3. After training, models were evaluated via Wekinator’s evaluation tool (accessible via the GUI) which computes cross-validation (CV) with 10 folds and also returns a TA score. However, Wekinator provides no evaluation tool for DTW models. Therefore, we devised our own evaluative method via recording the prediction frequency of DTW model output in response to a running model input/performed gesture. WEKA was also used to gather further evaluative metrics about model accuracy for consideration; Wekinator is built on this framework, so we used the corresponding models in WEKA and run evaluations under the same conditions (e.g., 10-fold CV).

#### 3.4.3. Running Ml Models in Wekinator Using Historic Data

We recorded newly performed instances of gestures 1–3 (using conditions stated in Section 3.4.2) to see if model accuracy was impacted when using historic gesture data. We imported such historical gesture data into Max 8 and used an array to read the data. The array then communicated historic gesture data to Wekinator for respective models to make predictions (see Figure 1 for an illustration of this pipeline).

#### 3.4.4. Optimising Trained Ml Models in Wekinator

The optimisation process involves arbitrarily modifying model parameters in Wekinator (manually) and observing model behaviour (see Table 2 for available models and their default parameters and model parameters for optimisation via Table A7, Table A8 and Table A9). Wekinator’s built-in model evaluation tool will be used to evaluate models during this process.

#### 3.4.5. Ml Model Example Length within Wekinator

Each gesture was assigned c.4000 data examples per class to train continuous and classifier models at a speed of 10 ms per example. However, other example sizes (starting from c.500) were also used to see if example size affects prediction accuracy. The DTW model has no GUI to show the user how many examples have been recorded; we use c.300 samples (over 2 example instances) to train each model across gestures 1–3. We also investigated other example sizes for the DTW model, using up to a total of 5 examples (where each example contains c.150 samples). See Table 2 for a detailed list of default model parameters (as described in Wekinator) when training each model across all gestures. The Wekinator model evaluation function was used for each model type over all gestures. However, Wekinator does not include the same tools for evaluating DTW models. Therefore, independent tests for accuracy were conducted for the DTW model (i.e., measuring model prediction behaviour over time).

## 4. Results

In this section, we report model prediction accuracies (for all model types) across performance gestures 1–3 and conditions that may affect such accuracy. In Section 4.1, we report model evaluation metrics (Wekinator/WEKA) and general observations returned when performing a static version of gestures 1–3 (all model types). Then, Section 4.2 presents evaluative metrics and model behaviours for onset models (where the gestural onset is included during training). Next, Section 4.3 reports the accuracy of supervised models in Wekinator when classifying historic gestural data. Following this, Section 4.4 reports the impact of model example length on model accuracy. After this, Section 4.5 presents observations on data post-processing/data type on model accuracy. Then, Section 4.6 reports observations when optimising parameters of all ML models in Wekinator (gestures 1–3). Lastly, Section 4.7 discusses how new instances of the music gestures investigated in this study can be classified via supervised learning (in Wekinator).

### 4.1. Model Evaluations (Static Models)

In this section, we investigate the best and most accurate methods when performing gestures 1–3, in real-time, statically and when training all respective static models. We discuss the onset models of these gestures in Section 4.2 and compare them to static models observed in this section. All models in this section use c.4000 examples per class (except the DTW model, which uses c.300 samples over 2 gesture examples).

#### 4.1.1. Continuous Models

Across all three static gestures, the NN was consistently demonstrated to be the most accurate model (see Table 3 for a full list of continuous model accuracies). Noticeably, gesture 1 reports the highest static gesture type model accuracies of all the gestures trained. However, gesture 2 is the highest static gesture type of the EMG gestures used. This is because gesture 2 is the least nuanced gesture of the gestures using EMG data and data variation, within the training dataset, is empirically lower than in gesture 3. We notice that the returned CV and TA accuracies are identical across all gestures; this is because data variation is extremely low amongst all gestures because of the nature of the gesture type (static). As a result, this may cause overfitting and a less effective generalisability indication offered by the CV score.

**Figure 5:**
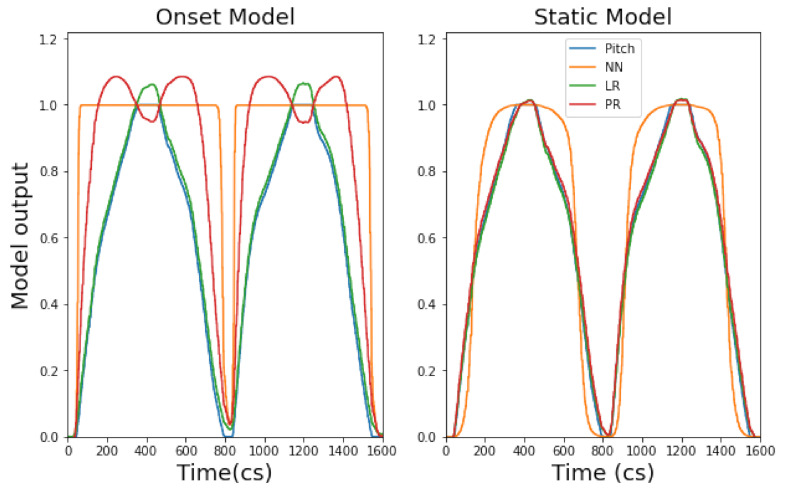
Graph showing mapping behaviour of NN, linear regression (LR) and polynomial regression (PR) onset models when performing gesture 1 (measured via Euler angle pitch and using c.4000 examples per class) over a period of 16 s.

**Figure 6:**
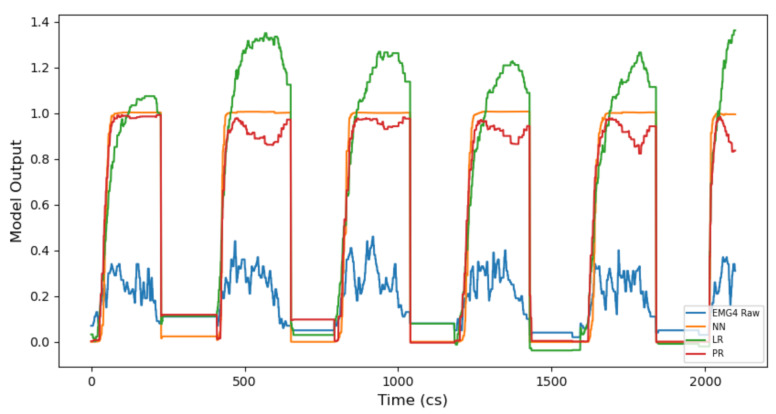
A graph displaying continuous (static) model outputs and their activity during a performance of gesture 3 (ii) over a period of 20 s.

It is also apparent that mapping behaviours are different between NN, LR and PR models. Continuous model results demonstrate that NN and PR models apply curvature to the mapping of input gesture data, whilst the LR model applies a mapping which is linear. When looking at the continuous model output of gesture 1, via the right hand side of Figure 5 (static), it is clear that the LR model maps the input signal and movement of the gesture closely. However, the NN creates a bell curve-like mapping and the mapping from the PR model is slightly more rounded and less linear than the LR model. Interestingly, when comparing the mapping behaviour of continuous model outputs for EMG gestures (i.e., gestures 2 and gesture 3), we still see the same behaviour. We see that the NN retains the same curvature behaviour, the LR still applies a linear mapping and the PR applies more mapping curvature to the input signal than the LR but less than the NN (e.g., see Figure 6).

Perceiving this difference between the NN, PR and LR models is important for interactive music practice. This is because the rate of data increase will create a disparate musical output when applying DSP to an audio signal (in musical terms, this is referred to as attack time—discussed more in Section 5). The number of examples for continuous model input, across all three performance gestures, can be seen in Table 3. Data regarding the strength/amplitude of gestural movement for static continuous models (using c.4000 examples per class across gestures 1–3), during training, can be found in Table A1.

#### 4.1.2. Classifier Models

Looking at Table 4, we see classifier models used across gestures 1–3 perform optimally (CV/TA of 100%) in most cases. This is surprising but may be indicative of overfitting, given the very low variation present in the training dataset of static models. However, we can also observe that the DS algorithm used with gesture 3 performs the poorest, compared to all other classifier models. The DS is thought to be less accurate because it uses a single attribute to classify datasets; therefore, it is considered a weak learner [48]. Gesture 3 uses five classes and eight inputs, therefore the single attribute used by the DS (i.e., decision threshold) would be severely impacted; due to an increase in data volume when making predictions using a number of classes >2.

Interestingly, we see that the k-NN consistently reports a higher root relative squared error (RRSE) score (via both CV and TA) over all gestures and classification models, with the exception of the DS model of gesture 3; meaning how well the model would perform using a simple predictor (average of actual values). This elucidates that, on average, the k-NN model provides slightly less predictive accuracy across all models used, regardless of the model’s optimal CV/TA scores, and the DS is significantly less accurate. Within Table 4, the CV and TA scores are identical because of the low variation present within the training datasets; as a result, the CV and TA processes derive the same conclusions. Therefore, the generalisability indication that CV usually gives is questionable, here, where static models are concerned.

We also see that particular models make correct classifications earlier (and later) than other gestures during performance. In this sense, model classification is staggered according to stages within the signal. With gesture 1, we see that the ABM1 and DT models are first to achieve a classification of 2 (when the arm is raised), followed by the k-NN and DS (unison), then the SVM, followed by the NB. The inverse of this order is true when the signal returns to a classification of 1 (i.e., lowering the arm).

Gesture 2 sees the NB model predict class 2 (activation) of this gesture first, followed by the ABM1 and DT (unison), then k-NN and DS models (unison) and, lastly, the SVM. The inverse of this classification order is also somewhat true when the gesture returns to a classification of 1 (hand resting); the difference is that the k-NN classifies the gesture (class 1) after the SVM, ABM1, DS and DT.

Gesture 3 shows that the worst performing static classifier model, regarding correct classification during performance, is the DS. This is promising as it shows that the accuracy metrics returned from Wekinator and WEKA are indicative of real-world application performance. We see a very promising performance of most models for this gesture. In terms of prediction speed via this static classifier model, we see that (on average) the SVM is consistently first to make a correct classification and the k-NN is consistently last; whereas, on average, ABM1 and DT models are ahead of the k-NN for prediction speed, but before the SVM. The NB model is very erratic in stabilising a correct prediction. The number of examples for static classifier model input, across all 3 performance gestures, can be seen in Table 4. Details regarding the performed amplitude of static classifier models (using c.4000 examples per class over gestures 1–3) during training can be found in Table A3.

#### 4.1.3. Dynamic Time Warping

DTW model output data taken for gesture 1 suggest that the model is effective in gestures with low variation data (IMU). However, we also see promising DTW prediction results for gesture 2. Lastly, gesture 3 shows promising DTW behaviour for prediction but is less stable than gesture 2 (also using an EMG dataset); this is because gesture 3 is highly nuanced and uses all five digits of the hand to execute it. Therefore, there is overlap between some muscle groups when classifying fingers—particularly when differentiating fingers 3–4 and 4–5. Please refer to Table A5 to see the recorded values for gestural strength when training static DTW models (using c.300 samples over two gesture examples) across gestures 1–3.

**Figure A1:**
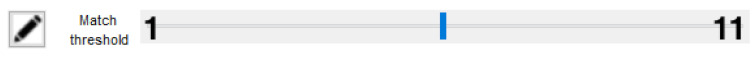
A scale we designate for the DTW model GUI in Wekinator, where each point in the scale is accessible by clicking the horizontal line, incrementally, with a mouse. The scale ranges from 1 to 11 accordingly (via Windows 10 operating system). The blue line shown on the scale is point 6.

One core weakness of using the DTW model is concerned with the GUI. When using the GUI to record samples and train DTW models, users are unable to know how long training samples are. This is because the metric does not appear in the GUI (unlike classifier and continuous models). The GUI ‘threshold’ for the DTW model is also arbitrary; allowing the user to alter model prediction sensitivity. However, a threshold sensitivity metric is unavailable via the GUI—therefore, we designed a metric/scale to navigate this problem (see Figure A1).

### 4.2. Model Evaluations (Onset Models)

In this section, we investigate whether onset model types are as accurate as scores returned for static model types, across all gestures. All models in this section also use c.4000 examples per class (except the DTW model, which uses c.300 samples over two gesture examples).

#### 4.2.1. Continuous Models

Results from the onset continuous models show a striking difference between onset and static gesture type mapping behaviours. We identify two key observations: an NN shows to be the strongest onset continuous model and there is a trade-off between model accuracy and the speed at which a gesture is mapped.

Firstly, we see that an NN is the most accurate onset model for gestures 1–3 (across TA/CV scores) where the LR/PR onset models always perform equally well. Secondly, we find that the onset models always reach their peak amplitude (1.0) much faster, across all gestures, than their static model counterpart. This is significant for music practice as it means a mapped music output will sound audibly different when deciding whether to include, or omit, onset gestural data (more on this in Section 5.3). Additionally, we see that including gestural onset with IMU models (i.e., gesture 1) affects the mapped shape of all model outputs (NN, LR, PR); compare the continuous onset and static mappings of gesture 1 in Figure 5. This is very interesting, as it will directly affect the music mapping process (discussed in Section 5.3). However, we do not see the same mapping behaviour for EMG gestures; this may be due to the higher level of noise within EMG datasets compared to IMU datasets.

When looking at the onset continuous model output of gesture 3 (ii), we see that the NN and PR models attempt to map the gesture with some curvature to the input signal. However, we see that the LR model is very erratic during mapping. This is interesting, as it represents a key difference between both the LR and NN/PR models when including gesture onset in the model training process. It is also significant because the findings can affect the decision to include onset gestural data when choosing continuous models for interactive music composition. Refer to Table A2 to see the gestural strength values recorded within onset continuous model inputs (using c.4000 examples per class) across gestures 1–3.

#### 4.2.2. Classifier Models

Regarding model accuracies for gesture 1, we see that the KNN, ABM1, DT and DS models are the strongest performers, where we note an accuracy of >99.6% for such models; however, we do also see that (alike static classifier models) the k-NN returns a higher RRSE over all models, suggesting the slight unreliability of the k-NN model above all models during prediction.

Gesture 2 shows that all models return an optimal score across CV and TA (100%) except the NB model (99.42%). The lower CV score for the NB is further corroborated by an unusually high (thus unreliable) RRSE score for the NB model (15.29%), followed by the k-NN (0.0021%).

Upon observation of gesture 3, we see all models except the NB and DS perform with high accuracy; the k-NN, ABM1 and DT models report >99.42% accuracy (CV and TA) and the SVM reports 94.29% (CV) with 94.33% (TA). The RRSE score for the k-NN is higher above all models except for the DS, the latter returning a high RRSE score of 86.50%; elucidating respective unreliability of the k-NN and DS across all models.

Similar to continuous models, it is apparent that there is a compensation between model accuracy and the speed at which the gesture is detected with onset classifier models. We see that gesture 1 follows a structured order of prediction speed between all models. When achieving a class 2 prediction (i.e., raising the arm above the head), the k-NN, ABM1, DT and DS models are first to make a correct classification, followed by the NB and SVM models. Interestingly, a clear pattern is formed when returning to class 1 (i.e., lowering the arm), which is very similar to the pattern observed when achieving a class 2 prediction. The worst onset model metric accuracies returned from Wekinator, for gesture 1, were the SVM (CV=99.42%) and NB (CV=99.53%). This is very interesting, as we find that the SVM and NB models consistently make predictions last, compared to all other onset classifier models.

Where Gesture 2 is concerned, the onset classifier models show a prediction in unison, except the NB, which has a maximum delay of 400 ms when making a correct classification. This is particularly interesting, as the NB model was reported to be the worst performer for gesture 2, with a CV score of 99.42%, thus corroborating the objective metric (returned from Wekinator) in practice.

When looking at a performance of gesture 3, we can see that the first (0–401 cs), second (401–801 cs) and fourth iteration (1201–1601 cs) of the gesture are detected earlier by an onset classifier model (k-NN) in comparison to a static model (k-NN). However, we also observe that an onset model is less accurate than a static model when looking at the returned accuracies of a static classifier model and onset classifier model for gesture 3. In particular, we see that the NB and DS models are the worst performing classifier models for gesture 3, regardless of whether the onset of the gesture is included or not. Model accuracy metrics are further corroborated with gesture 3 when individual model prediction behaviours are observed.

Therefore, we observe a speed of gesture detection vs. model accuracy trade off over all three gestures, which include the onset of a gesture during training. See Table A4 for gestural amplitude values within onset classifier model inputs (using c.4000 examples per class) across gestures 1–3.

#### 4.2.3. Dynamic Time Warping Models

We note very interesting results when we compare static and onset models, across all gestures, for the DTW algorithm. As a DTW model relies on the prediction of a gesture over time, it is especially important to investigate whether onset detection plays a significant role in gesture prediction accuracy.

**Figure 7:**
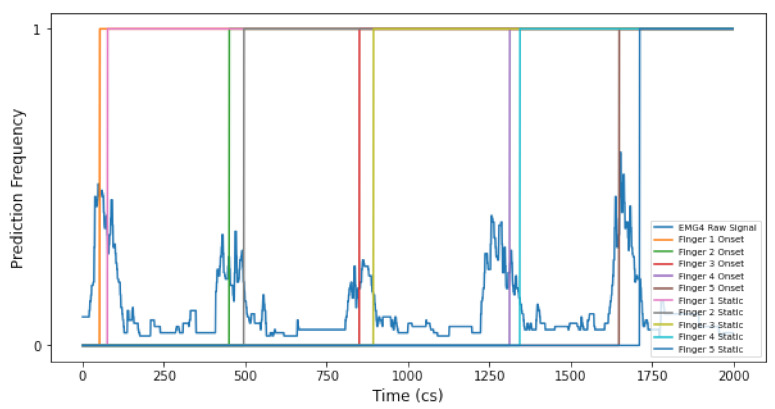
A plot comparing the enumerated prediction frequency of both DTW static and onset models when predicting the performance of gesture 3 over 20 s, using all five digits (i–v) of the right hand. A prediction frequency of ’1’, when each digit is used to perform (over onset and static approaches), is optimal and shows a correct prediction instance by a DTW model.

Results returned from using the gesture 2 DTW onset model show faster prediction times than static models (i.e., where the onset is omitted during training). Similarly, when looking at Figure 7, we can see how the DTW onset model predicts each finger of gesture 3 earlier than a static model. Therefore, this has clear implications on model type choice in the context of music performance and composition (i.e., static or onset), due to the temporal nature of the artform. This finding can be used to inform literature in the field of IML practice, within music, which states that DTW algorithms are inadequate because they make a classification at the end of input [16].

However, we see contrary results for gestures using IMU data only (i.e., gesture 1). As the IMU dataset for gesture 1 is linear and variation is low, we find that training a DTW onset does not work as effectively as when using EMG data with onset models; in this sense, we find that using gesture 1 with onset DTW models does not make a noticeable difference in prediction speed. Please see recorded gestural strength values for onset DTW models (using c.300 samples across two gesture examples), over gestures 1–3, within Table A6.

### 4.3. Running Supervised Models with Historical Gesture Data

In this section, we study if historical gesture data can be used to create successful predictions with previously trained models (using onset and static versions with c.4000 examples per class) across all gestures. To do this, we use new instances of gestures 1–3 as input data for trained models in Wekinator and observe behaviour via offline analysis.

#### 4.3.1. Continuous Models

Continuous models show interesting results where NN onset and static models are concerned, when analysing the behaviour of historic data predictions.

Gesture 1 shows that the time for the onset model output to reach maximum amplitude (i.e., 1.0) is faster than static model output. However, we also observe that the shape of the NN and PR onset model output changes significantly to become much more rounded. This is significant because we also see this for gesture 1 real-time data results. Thus, new instances of the gesture behave as we previously observed and expected. We see the same behaviours when using two new static (fixed) performances of gesture 1 (i.e., one recording at the location of class 1 and the other at class 2), as the real-time continuous model performances.

Using historic data with gesture 2 shows the same mapping behaviours as when using real-time data. It also shows that using historic data, including the onset of the gesture, affects the prediction of static models. This is because the static model is not expecting the variation present within the gestural onset. The PR model is adversely affected, but the NN and LR models behave as expected (i.e., as when the onset is omitted). Conversely, we see that historic data omitting the onset of the gesture (i.e., a ’static’ artificial performance) do not negatively affect the prediction accuracy of onset models (trained with onset gestural data).

When an example of both an onset and static performance of gesture 3 (i) is introduced to a continuous model trained with static and onset gesture types (i.e., models trained with either examples of the onset of a gesture 3 (i) or without the onset of gesture 3 (i)), we see that the NN is most accurate when a static performance of gesture (i) is used. In this sense, NN continuous models are less accurate when mapping the performed onset of gesture 3 (i) only. This is because data variation is higher with onset models. These findings are significant because it shows that NN continuous models are stronger to use with artificial static gesture performances only, rather than onset performances of gestures using post-processed EMG data. Furthermore, we also see that LR and PR onset and static models behave in the same manner when mapping both the onset and static performance of gesture 3 (i), when using historic data to do so.

#### 4.3.2. Classifier Models

Where gesture 1 is concerned, we see that onset classifiers always make correct classifications faster than static models. Moreover, we see that all gesture 1 onset models are more accurate compared to static models when using historic data, where the NB static model under performed quite noticeably. We notice that previously trained gesture 1 onset/static models display these same behaviours, during prediction, when using a static performance of gesture 1 (i.e., capturing data so gestural onset is not included).

Looking at gesture 2, we observe that the SVM and k-NN onset models are unreliable when used to predict a performance of gesture 2 (where the onset is included). This is very interesting as the SVM is also the least reliable of classifier models, regarding prediction efficacy, when using real-time data (see Section 4.1.2). However, all static classifier models are accurate. We also notice that some onset classifiers (DT and ABM1) are 20 ms faster than static models to make a prediction; however, this is a marginal difference. Moreover, we see no conclusive results when comparing onset and static models, using an artificial static performance of gesture 2, to make a correct prediction.

Gesture 3 shows that all static models are more accurate than onset models. However, onset models make a prediction slightly faster than static models. When using a static performance of gesture 3 (artificially) we see that, overall, static and onset models predict equally well. This means that future performances of gesture 3 can include or omit the onset of a gesture, and the trained models (static/onset) will make reliable predictions.

#### 4.3.3. Dynamic Time Warping Models

Gesture 1 shows that the DTW onset model is much faster than the DTW static model to make a prediction. However, it is worth noting that both models used different sensitivities, via the DTW GUI, in order to elicit a correct prediction according to the behaviour of the model(s); the onset model used a DTW setting of 10 and static DTW model used 10.5 (see our scale via Figure A1). We cannot successfully test an artificial performance of gesture 2 with DTW models using historic data; they cannot operate when a gestural onset is not included, as the algorithm needs to see a clear pattern over time with higher variation in the dataset. However, this is not the case during Section 4.1.3, as we train a static DTW model and then perform gestures 1–3 with the onset included.

Regarding gesture 2, we also notice that the DTW onset model is faster than the static to make a prediction (when gesture onset is included).

Concerning gesture 3, onset DTW models predict an onset performance with higher accuracy than static DTW models. This is interesting, as real-time onset performances of gesture 3 are also predicted with better efficacy when using onset DTW models (see Figure 7). However, we also find that static DTW models predict gesture 3 (inclusive of the onset of the gesture) 10 ms faster than the onset DTW model. This is surprising, because Figure 7 shows that real-time gesture 3 data are predicted earlier by an onset DTW model. This may be explained in relation to the ‘threshold’ GUI used by a DTW model, whereby the threshold allows the user to set prediction sensitivity. As mentioned with gesture 2, we also cannot test a static performance with this DTW model. Therefore, it is likely that the DTW threshold was set to be more sensitive for the static DTW model than the onset DTW model (see Figure A1 to see the threshold used by the Wekinator GUI for DTW models).

When testing static gesture data with both static/onset DTW models, it became obvious that static gestural data are not at all suited for DTW models. This is because the algorithm detects movement in the data over time; if there is no movement—i.e., a static gesture is used—then the DTW model is wholly ineffective. This is important because it allows us to be aware that static behaviours, in music, are not suitable when using DTW models with historic performance gesture data.

### 4.4. Number of Model Examples vs. Model Accuracy

In this section, we ask if the number of model examples used per class, across gestures 1–3, affects model accuracy (returned via the Wekinator evaluation tool). We do this for both static and onset model types.

#### 4.4.1. Continuous Models

Concerning gesture 1, we see that a greater number of examples (c.4000 per class) improve onset continuous models. Regarding gesture 2, we see that a greater number of examples (c.4000 per class) reduce LR/PR static models accuracy, but the NN model is unaffected. Interestingly, however, the accuracy of onset models is unaffected as more examples are introduced to gesture 2. Where continuous models are concerned for gesture 3 (both static and onset model types), we see that the LR and PR models become less accurate between c.500 examples per class and c.4000 examples per class. Interestingly, however, the NN retains an optimum measured accuracy.

#### 4.4.2. Classifier Models

**Figure 8:**
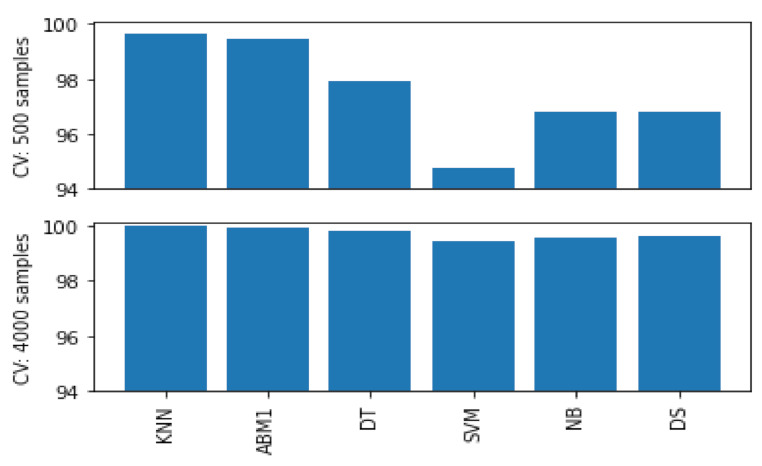
Bar plots showing cross validation (CV) accuracies returned from Wekinator when assessing how increasing number of examples per class (c.500–4000) affects all gesture 1 onset classifier models.

Regarding gesture 1, we note that a greater number of examples (from c.500–4000 per class) improve onset classifier models accuracy (see Figure 8), but static models remain unaffected. Gesture 2 shows that a greater number of examples also improve onset classifier models, but static models remain as accurate. Regarding gesture 3, static classifiers show that the NB model becomes less accurate and the DS increases in accuracy, as more examples are introduced. This is interesting because such accuracy scores are in response to an increase in variation within the dataset. Moreover, all onset classifier models uniformly decrease in accuracy as more examples are introduced to gesture 3.

#### 4.4.3. Dynamic Time Warping Models

Lastly, we observe that the DTW model is less accurate when given more examples, across all gestures, using a maximum of 5 examples to test this. This may be due to increasing the variance within the model dataset and increasing the noise level when adding more examples to the model. However, we note that static DTW models are the best performing version of the DTW model when fewer examples are given, compared to the onset DTW model with the same number of examples. Interestingly, we also see that a greater number of examples (i.e., 5) affect prediction speed across both static and onset DTW models; in this sense, prediction speed is faster when fewer examples are used. Lastly, we also note that the threshold via the Wekinator GUI, when using the DTW model (see Appendix D, Figure A1), is integral in affecting prediction accuracy, regardless of the number of examples used; it must be idiomatically optimised, as we observe that the threshold needs to be set much higher when a greater number of examples are introduced to the model. These findings corroborate literature in the field, which states that DTW models are operable when given fewer examples to make a template [16].

### 4.5. Impact of Data Type and Post-Processing on Model Accuracy

In this section, we investigate whether data post-processing improves model accuracy, juxtaposed to not using post-processing methods with gestures in music. We do this by reporting all model accuracies, before post-processing is applied vs. after post-processing is applied, to all static and onset gestures (c.500–4000 samples per class). We also observe whether data type used (IMU) affects model accuracy. We find that using Euler pitch data with gesture 1 (compared to using y-axis acceleration data [49]) does not improve static continuous model accuracy when using c.500 examples per class (i.e., 0.0 and 1.0). However, using Euler pitch data does improve static continuous model accuracy by 99% (TA and CV), when using c.4000 examples per class, for LR and PR models. Albeit, the NN does not improve. Onset continuous model accuracy improves by 14.28% (CV) across LR and PR models, when using c.500 examples per class, but the NN remains unaffected. When using c.4000 examples per class, however, the NN accuracy increases by 16.66% (CV and TA) and LR/PR models increase by 22.22% (CV and TA). Static classifier models do not improve when using c.500–4000 examples per class, except the DT model, which improves by 0.19% (CV - employing 500 samples per class). Most onset classifier models see a reduction in model accuracy when using between c.500–4000 examples per class; only SVM and DS models improve between a range of 0.01% and 0.68% (CV). DTW model accuracy prediction is greatly improved (using pitch data) across all sample sizes and gesture types.

Regarding gesture 2, static classifiers show the best improvement when applying post-processing to the model dataset, using c.500–4000 samples per class (see Table 5); we see SVM and DS models consistently improve their accuracies more than other static classifier models. Onset classifiers also show an improvement overall when using post-processing, albeit less than static models. However, the NB becomes worse when applying a post-processing stage to the EMG dataset when using only c.500 samples per class; the NB model improves when using c.4000 samples per class. Similar to static classifier models, we also see the SVM and DS models improve the most when using onset classifier models and c.4000 samples per class. Continuous models show that every model improves by >89.65% (TA and CV) when post-processing is applied, where continuous static models improve more than onset models. However, the NN improves the most in all experimental cases; i.e., when using both onset and static models and c.500–4000 samples per class. DTW model prediction is also more efficient when using a post-processing stage with gesture 2 across all sample sizes and gesture types.

Regarding gesture 3, we also see that all onset and static classifiers improve in accuracy, when using post-processing with c.500-4000 samples per class. In particular, we see SVM and NB onset classifier models improve by >40% (TA and CV) and static SVM and NB models improve by >50.48% (TA and CV). All continuous onset models also improve when using c.500–4000 samples per class, with an increase in model accuracy of >41.66% (TA and CV); however, static continuous models increase by >81.48% (TA and CV). Lastly, the DTW model is also far more accurate when using post-processing treatment with gesture 3 across all sample sizes and gesture types.

### 4.6. Optimising Supervised Learning Models for Gestures 1–3 in Wekinator

In this section, we ask whether all models in this study can be optimised across gestures 1–3. We do this via changing model parameters, manually, through gestures 1–3 and collecting model accuracy scores (via Wekinator GUI), as well as observing model behaviours offline (see Appendix E, Table A7, Table A8 and Table A9, for a list of model parameters used to optimise all models. Moreover, see Table 2 for default model parameters).

We find that gesture 1 SVM onset classifiers, using both 500 and 4000 samples per class, can be optimised across all kernels (linear, polynomial and radial basis function (RBF)). This is done via increasing the complexity constant (1–1000), exponents (2–10) and gamma values (0.01–1000) across all SVM onset kernels. However, we do see that DTW models can also be optimised. Where DTW onset models are concerned, we see a difference between real-time prediction (via model parameter matches computed continuously) and offline prediction (i.e., via parameter matches computed when running stops). For real-time prediction, we see that when using the model parameter ‘downsample so examples have max length of’ 10 and ‘continuous matches use a minimum length of’ 5, then reducing match width size from the default value of 5 to 1, the time taken for a correct prediction to occur becomes much faster. We also see that model performance improves when parameter ’downsample by constant rate’ is used and a match width is reduced from 5 to 1. For offline prediction, we see that the model can be optimised when ’downsample by constant rate’ of 5 and ’continuous matches use a minimum length of’ 5 is used, paired with a lower match width (i.e., 1). We also see the same behaviour when a ’continuous matches use a min length equal to the shortest example’ model parameter is used with a lower match width size (again, 1). Regarding DTW static models for gesture 1, we see optimum performance for real-time prediction when ’downsample by constant rate’ of 10 and ’continuous matches use a minimum length of’ 5 is used. For offline prediction, best results are when ’downsample so examples have max length of’ 20 and ’continuous matches use a minimum length of’ 10 are used.

Concerning optimisation for gesture 2, we see that SVM onset classifier can also be optimised using 500–4000 samples per class, across all kernels. Like gesture 1, this is done via increasing the complexity constant (1–1000), exponents (2–10) and gamma values (0.01–1000) for each kernel. However, there is one crucial finding; when we do not use lower order terms for the SVM onset model using a polynomial kernel, we cannot optimise the model. Instead, using lower order terms adversely affects the SVM model. We also find that DTW models can be optimised. Regarding DTW onset models, for real-time prediction, we see that higher ’downsample so examples have max length of’ values, paired with higher ’continuous matches use a minimum length of’ values, make DTW onset models more accurate. We note that in particular, the onset of the gesture is detected faster. For offline models, we see that the DTW onset model is most accurate when ’downsample so examples have max length of’ parameter is used with higher values (≥50), ’downsample by constant rate’ parameter is also used with higher values (≥50) and ’don’t downsample’ parameter is used—but always paired with ’continuous matches use a min length equal to the shortest example’. However, DTW static models can also be optimised. We find for real-time prediction that a lower ’downsample by constant rate’ value, paired with a lower ’continuous matches use a minimum length of’ value, provide the best performance. This improves when a lower match width is used. For offline prediction, the best setting is when we use the ’don’t downsample’ parameter alongside a value of 5 for the ’continuous matches use a minimum length of’ parameter.

**Figure 9:**
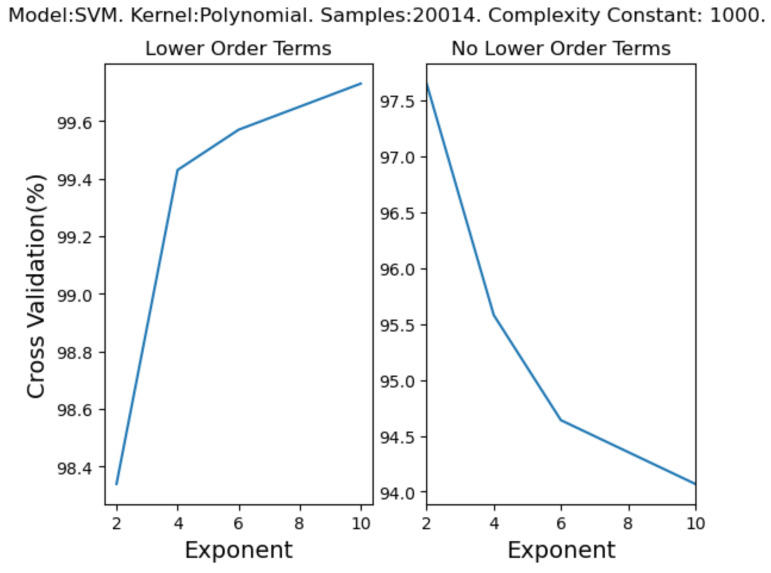
A plot of comparison showing gesture 3 support vector machine (SVM) onset classifier model accuracy (via CV) when using 20,014 examples, and a polynomial kernel, to optimise the model through using both lower order terms and not using lower order terms. Exponent value of the kernel complexity constant =1000.

Gesture 3 shows that ABM1 and SVM onset classifier models can be optimised (between c.500–4000 examples per class), as well as DTW onset/static models. We see the same results taken for gesture 2 in this respect (see Figure 9); this is not surprising, as both gestures use EMG data (with the same post-processing treatment) to train all models. Additionally, we find that DTW onset real-time models perform best when ’downsample so examples have max length of’ is set at 10 and when ’continuous matches use a minimum length of’ is set at 5; each being a low value. We also see that reducing the match width default value from 5 to 1 further increases accuracy. Offline prediction is best for the DTW onset model when ’downsample so examples have max length of’ value 20 is used, with ’continuous matches use a minimum length of’ value 10, is also used. We further see that DTW static models provide the same results as the onset model, as above.

### 4.7. Reflections on Model Choice When Classifying Performance Gestures 1–3 in Wekinator

In this section, we reflect on results found between Section 4.1, Section 4.2, Section 4.3, Section 4.4, Section 4.5 and Section 4.6 and discuss how our findings could be used to classify new instances of piano performance gestures used in this study (using EMG/IMU data and the same study conditions). This research focus is important because it is a desirable aim within the field of IML and music [12]. In this investigation, we find consistent results for model accuracies (across all gestures used and performance gesture types: static and onset) and mapping behaviours; we see this when looking at how our trained models process and respond to real-time and historical gestural data. Thus, we take these findings to suggest that IML practitioners can build their own instance of the performance gestures used in this study (based on piano practice) and determine model accuracy/behaviour, when sonically representing such gestures via three types of algorithms: classifier, continuous and DTW. It is important to note that the gesture–algorithm relationship is vital because pairing gesture characteristics with algorithm characteristics (e.g., model output type) can alter creative expression [17]. Therefore, we suggest that new instances of the three gestures used in this study may be built for interactive music (piano) practice (using the same methods cited in this research) via the following process:

1. Pre-process, post-process and select the best data type for the chosen performance gesture from this study; using a MAV function for a gesture reliant on EMG information and Euler pitch data for a gesture measuring a linear upwards vector (i.e., elevation); refer to Section 3.3 and Section 3.3.1 regarding this process.
2. Inform chosen gesture type by addressing this question: is the perceived onset (attack time) of the gesture vital to the particular music process and aims? Answering ’yes’ will direct the practitioner to record your chosen gesture in Wekinator, including the onset of your gesture. Answering ’no’ will direct the practitioner to record the gesture statically, without the onset; refer to Section 3.2.1 for a description of both gesture types.
3. Establishing chosen gesture type then informs model choice. We suggest that there are two model types to choose from: prediction via generating a fixed music event or live music event. A fixed music event will require a classifier model, whereas a live music event will require a continuous or DTW model. However, the choice between a continuous and DTW model, for a live event, will have an important consequence; choosing only a continuous model for a live music event will provide the user an additional layer of musical information regarding the disparate mapping behaviour of each algorithm (i.e., NN, LR and PR—see Section 4.1.1 for mapping behaviour. The direct implication of this concept on music is discussed in Section 5).
4. Next, the practitioner should choose their particular algorithm within the model type chosen (refer to Section 4 to inform this decision through observing model accuracy).
5. Record the chosen gesture (from this study) using the Wekinator GUI. Refer to Section 4.4 when deciding on number of examples for best practice regarding the chosen model(s) type.
6. Next, optimise the model(s) (refer to Section 4.6 for information regarding the optimisation of classifier and DTW models).
7. Train model(s) and then run Wekinator for the composition process.

## 5. Interactive Music Composition

### 5.1. Model Output: Interactive Music Composition

In this study, we observed how different ML models (in Wekinator) behave when predicting performance gestures (based on a focused musical context for each gesture—see Section 3.2) and their subsequent potential to inform interactive music practice. In particular, we investigated how several ML models can disparately affect IML processes (in the studied contexts) due to their different, underlying, algorithmic behaviours. In this sense, if ML application becomes increasingly integral to future interactive music practice, it is vital to understand how ML models offered through popular IML softwares (i.e., Wekinator) can be used accordingly—here through augmenting piano practice. Moreover, we offer a discussion of how the studied model outputs, derived from the observed gestures, could be used within interactive piano music practice, as follows:

#### 5.1.1. Continuous Models

In the context of this study, we have seen how continuous models are useful for stimulating music parameters in real-time, during gestural performance, due to their nature. Each model has also been shown to process model output mapping (mapping is an integral part of interactive music composition and computer music. It means to use a transformed parameter to control or affect another parameter [50]. Here, a model output to affect a DSP process (e.g., delay time, reverb, etc) of an audio signal) disparately. Observing this behaviour is significant, as interactive music is composed through the multi-parametric manipulation of DSP to sonic material [51]. Therefore, the mapped output (shape) from each individual model output will provide a corresponding individual and unique music output. This can be demonstrated, for example, when establishing a 1:1 relationship between a continuous model output (NN, LR and PR) and a sine wave; where each model output will affect sonic output, disparately. An NN would smoothen the movement between each frequency cycle and both the LR/PR would make the behaviour more turbulent. However, it should be noted that some DSP events welcome turbulent movement within data (i.e., granular synthesis) and others a more rounded (smoother) movement (i.e., delay lines), due to their respective music aesthetic. Thus, these observations can be applied to interactive music piano practice when augmenting music material; e.g., playing a chord and lifting the arm (i.e., gesture 1) to create such a mapping, using the different mapping behaviours offered by the NN, LR and PR. An aural example of the consequence of model mapping behaviour, within interactive piano music practice, is shown in Section 5.3.

#### 5.1.2. Classifier Models

Classifier models can be used to successfully differentiate stages of gesture performance (i.e., through using multiple classes/labels). This is useful because a single performance gesture can activate several predetermined algorithms, at different stages of gesture performance, via a particular classifier model; useful within interactive music composition practice because it allows the composer to activate a fixed media (predefined) music output and allow the composer greater control over the process. This can be applied to piano practice when we look at gesture 2 in our study; resting and then extending the fingers, thus navigating between classes 1 and 2. However, the sonic output could be further detailed and processed by a method of convolution. This is done via using other datasets to stimulate the sonic signal during performance, once the algorithm (for each integer class) has been activated.

#### 5.1.3. Dynamic Time Warping Models

Like the classifier models, we see that DTW models can trigger a predetermined DSP algorithm. However, they cannot control parameters of the running algorithm after activation; this is because DTW models do not offer multiple classes. In this sense, a DTW model provides less layers of depth when representing a single gesture than a classifier model. Due to the fact that DTW models offer one-shot events, with no native parametric control available for DSP, they are suitable for music composition within a fixed DSP system/sonic environment. However, we have also seen how DTW models process gestural data faster than static models, suggesting that interactive piano music practice can be augmented when matching music material (aesthetics) to prediction speed. An example of using the DTW model within interactive music practice is when performing gesture 3 and augmenting the playability of traditional piano practice (via the ’tabletop’ piano, discussed in Section 5.2) through assigning a single note—or DSP process—to each finger. A DTW model is convenient in this context because it is inaccurate for a note to continuously ’fire’, when emulating how a traditional piano scale is played.

### 5.2. Ml Findings to Facilitate Novel Dmis: Gesture 3 as a Tabletop Piano

Gesture 3 was created in this study to use current techniques of best practice when processing EMG data and outline optimal ML methods when beginning to classify performance gestures within interactive piano practice. However, another aim was to facilitate the creation of novel DMIs. Using modern GIs to design DMIs is no novel concept in the field. However, the technology which drives them provides novel interactive opportunities. Modern DMIs rely on ML because of the complexity of biometric data (i.e., EMG) retrieved from the GIs which drive them [17,25,27]. Thus, if ML is an integral part in designing the modern DMI, then ML algorithms must be evaluated and considered for best practice when doing so; it can also stimulate a framework to inform future DMI design via ML models. Current literature has modelled DMIs on real instruments to focus on their pedagogical application [24], but seldom with compositional objectives. However, other literature designing DMIs have shown that understanding how we interact with acoustic instruments can better inform DMI action-sound mapping strategies [27]. We use our findings and introduce gesture 3 to facilitate traditional piano practice on the ’tabletop’. A video performance of our piano DMI, through using a static DTW model to perform gesture 3, can be seen here: finger classification from DTW (static) model during gesture 3 performance (https://www.dropbox.com/s/mexmyrhdk10az27/DTWStaticGesture3.mov?dl=0).

### 5.3. Ml Model Mapping Behaviour as a Creative Application within Music

**Figure 10:**
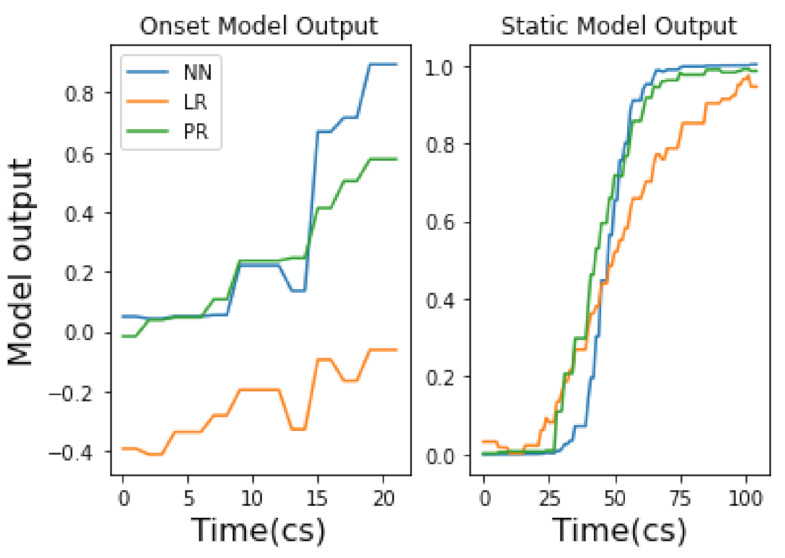
Two plots showing onset and static continuous model output of the NN, LR and PR models when performing gesture 3 (ii) over time (measured in centiseconds(cs)).

This study has elucidated that an understanding of algorithm mapping behaviour is of fundamental importance to interactive music practice, which is not possible to understand without looking at how ML algorithms process data at a quantifiable level. This is objectively evident when looking at how different continuous (regression) models in Wekinator disparately process a single performance gesture (compare how onset and static models map gesture 1 within Figure 5 and Figure 6 for static models mapping gesture 3(ii)). However, we also observe another key difference regarding mapping between onset and static continuous models for music. The rate at which the model output reaches its maximum value (i.e., 1.0) is disparate between continuous models trained with onset or static gesture types. This relationship is referred to as attack (attack time is defined as the time taken for an audio signal to reach full amplitude [52].) time within music practice. This relationship is most obvious when we see how a continuous model processes onset and static models of gesture 3 (ii). Looking at Figure 10, we can see how the continuous onset model output takes 23 cs (230 ms) to reach model output peak (i.e., 1.00) and how a static model output takes 92 cs (920 ms). Therefore, the static model takes 69 cs (690 ms) longer to reach its peak compared to the onset model, meaning that the onset model is more than half a second faster than the static model at mapping the model output, when trained with the inclusion of gestural onset data. This is important to note in music practice because it allows us to take advantage of this behaviour, creatively. You can hear an example of this discussion when we sonify (sonification is the process of representing data aurally, whereby the sound produced reflects the objective properties within the input data [53]) the model output of both a static NN model and an onset NN model with granular synthesis: onset performance (https://www.dropbox.com/s/sk1387t89mf3f8q/NN_Onset_Granular.aif?dl=0) and static performance (https://www.dropbox.com/s/cggthrj3annx247/NN_Static_Granular.aif?dl=0).

### 5.4. Using Historic Gesture Data within Music Practice

In this study, we investigated how using historic gestural data (of the three gestures studied) allows ML models (in Wekinator) to make very similar predictions as when using real-time gestural data. This is useful because literature has seldom explored how historic gesture data can be used within IML and interactive music practice, instead focusing on real-time applications [17,27]. Whilst the implementation of real-time systems may be obvious for IML and music practice, offline systems may be beneficial because they enable us to access external gestural information via the Internet. This is useful for the Internet of Musical Things (IoMusT) applications (based on the paradigm of the Internet of Things, the IoMusT refers to wireless networks of smart technologies which communicate and allow for inter-connectivity amongst the practical output of composers, musicians and people working within musical domains [54]) because recorded gestural information can be delivered to train supervised models. In this sense, desirable performance gesture information (captured by GIs) can be recorded (e.g., from virtuosic musicians) and used for training; a learner musician can then use the ML system to benchmark their playing against the trained model.

## 6. Conclusions and Future Work

In this study, we have shown that three focused performance gestures (for piano practice) can be used to provide unique composition opportunities within interactive music, by providing evidence for best model prediction accuracy (via type), differences in onset signal times and continuous model mapping behaviours. This is done via using a novel GI (Myo armband), a single performer, biometric datasets (EMG data) and evaluating all available ML models in Wekinator. When such findings are applied to interactive music practice, through the context of the study (gestures for augmenting piano performance), this provides evidence for unique musical devices; as heard/seen during Section 5.2 and Section 5.3). Regarding gesture representation, we can see that static models are more accurate than onset models across all the gestures—and model types—studied. However, it is also noticeable that there is an accuracy–prediction speed trade off. This is because onset models have been found to be generally faster than static models when making predictions, across all model types and gestures; albeit less accurate than static models. Therefore, we see that there are clear musical implications for how users represent our studied gestures when omitting or adding gestural onset (as discussed in Section 4.7).

When investigating model metrics, classifier models elucidated that all models except the k-NN and DS demonstrated promising performance for static gestures. However, the ABM1 and DT were the most commonly accurate classifiers across all onset gestures. We also see that the NN is the best performing continuous model (for both onset/static models) across all gestures. Additionally, we find that mapping behaviour of continuous models can be altered via specific model conditions. This is elucidated twice: when the disparate mapping behaviour of onset and static continuous models are compared (e.g., Figure 5) and when the signal attack time of all onset models is faster than in static models (i.e., predicting earlier), where this mapping behaviour is the same when using historical data with trained continuous models. Thus, these findings have the potential to affect interactive music practice, concerning the studied gestures, because they are aurally perceivable (see Section 5.3).

Additionally, we have seen how it is possible to optimise onset classifier (SVM and ABM1) and DTW models, across gestures 1–3, through modifying model parameters. Moreover, we observe that model example length can be studied to improve accuracy for classifier and continuous models; where more examples improve onset continuous and classifier models only, for our studied gestures. We have also investigated how historic data perform (regarding gestures 1–3) when introduced to previously supervised, trained, models; mimicking the behaviours and results seen for real-time models. Moreover, we also find evidence that current post-processing methods greatly improve the performance of the studied EMG performance gestures for use in interactive music composition practice. However, we also see that using a low variation, single vector, IMU data type (pitch) is suitable for good model performance and gestures which rely on IMU information (gesture 1, in this study). Furthermore, our results allow us to create suggestions for interactive music (piano) practitioners, when using our studied gestures, to create aesthetically interesting and efficient models.

Finally, we take our findings and apply them to build a novel case for interactive music practice, where we use gesture 3 to create a unique DMI—a tabletop piano. This DMI informs other work using ML and EMG interfaces in the field because we evaluate which models affect the playability of the DMI and similar DMIs. We also use current findings in best practice when processing EMG data to create highly nuanced gestures (i.e., all digits of the right hand within gesture 3) to drive a complex tabletop piano DMI. Such thinking could therefore affect how the ML process is considered (especially gesture representation) in the wider field when designing novel DMIs with EMG data.

Future work seeks to study gestures for interactive music practice with other instruments/musical material and compare supervised model behaviours; then, a wider framework for IML within interactive music practice may develop. In turn, this would allow us to query if unsupervised learning can be implemented when addressing the research problems investigated in this study. We also seek to establish a threshold for data variation before models can no longer be optimised. Also, we would like to investigate the automation of the model optimisation process. This study used a single performer during model training and testing in order to build a proof-of-concept and basis for wider investigation. Thus, in future work, we would like to investigate how our results can be informed by using multiple music performers to train models and how/if the studied gestures can be used in different music contexts. To add to this, we would also like to investigate the classification (in Wekinator) of more gestures within piano practice, in order to provide more musical contexts for their use. Furthermore, we are interested in using our findings to see if a library for gesture prediction can be built for music performance and the classification of new gestures. Additionally, we intend to use our findings regarding continuous model mappings (DSP) within different music performance situations (using our studied gestures 1–3) and by various music performers, in order to assess their wider suitability in music practice. We also seek to investigate how the Myo/EMG data can be used as part of a multimodal sensory system to capture performance gestures within Wekinator and if model performance is affected.

## Figures and Tables

**Table 1:** A table outlining key information from studies in music gestural recognition, as discussed in this section. The machine learning models used are: multilayer perceptron neural network (MPNN), hierarchical hidden Markov models (HHMM), decision tree (DT), hidden Markovian model (HMM), dynamic time warping (DTW) and recurrent neural network (RNN).

Study	Targeted ML Models	No. Gestures Used	Type of Gestures Used
Tanaka et al. [17]	MPNN and HHMM	4	All gestures user-designed in a workshop
Dalmazzo and Ramirez [24]	DT and HMM	4	4 fingers (left hand, index to ring fingers)
Ruan et al. [25]	MPNN	3	3 fingers (left hand - thumb, index, middle)
Di Donato et al. [26]	MPNN and DTW	6	5 activation gestures and 1 modulating gesture (left arm)
Erdem et al. [27]	RNN	6	6 gestures (exercises on guitar playing - both arms)
Dalmazzo and Ramírez [28]	HHMM and DT	7	Violin bowing techniques

**Table 2:** Available model types in Wekinator and their default settings.

Model Type	Model	Model Parameters and Their Default Settings	Model Output Range/Type
Continuous (Soft Limits, meaning the maximum model output (0–1) can be exceeded.)	NN	1 hidden layer. 1 node per hidden layer.	[0.0–1.0] (float)
	LR	Linear inputs. No feature selection used. Colinear inputs not removed.	
	PR	Polynomial exponent = 2. No feature selection used. Colinear inputs not removed.	
Classifier	k-NN	Number of neighbors (k) = 1.	[1–5] (integer)
	ABM1	Training rounds = 100. Base classifier = Decision tree.	
	DT	Model not customisable.	
	SVM	Kernel: linear. Complexity constant: 1.	
	NB	Model not customisable.	
	DS	Model not customisable.	
DTW	DTW	Matches computed continuously while running. Downsample so examples have max length of: 10. Continuous matches use a minimum length of: 5. Match width: 5. Match hop size: 1. Threshold for prediction set in GUI at scale value of 6 (see Figure A1)	Single fire

**Table 3:** Accuracy of static gesture continuous models (gestures 1–3) after training (post-processed), measured in root mean square error (RMSE), where 0 is optimal. Values in bold highlight the best performing model for each gesture (i.e., neural network (NN)). In the table, training accuracy (TA) and cross validation (CV) evaluation metrics are shown across all gestures. Gestural amplitude values, found within training data for static continuous models (using c.4000 examples per class), can be found in Table A1.

Gesture No.	Model	TA	CV	Class No.	No. Class Examples
1	NN	**0**	**0**	0.0	4006
	LR	0.01	0.01	1.0	4024
	PR	0.01	0.01		
2	NN	**0**	**0**	0.0	4021
	LR	0.02	0.02	1.0	4016
	PR	0.02	0.02		
3 (i)	NN	**0**	**0**	0.0	3911
	LR	0.05	0.05	1.0	4116
	PR	0.05	0.05		
3 (ii)	NN	**0**	**0**	0.0	3871
	LR	0.03	0.03	1.0	4136
	PR	0.03	0.03		
3 (iii)	NN	**0**	**0**	0.0	3997
	LR	0.05	0.05	1.0	4017
	PR	0.05	0.05		
3 (iv)	NN	**0**	**0**	0.0	3882
	LR	0.04	0.04	1.0	4155
	PR	0.04	0.04		
3 (v)	NN	**0**	**0**	0.0	3799
	LR	0.03	0.03	1.0	4243
	PR	0.03	0.03		

**Table 4:** Accuracy of static gesture classifier models after training (post-processed), measured as a percentage of correctly classified instances (where 100% is optimal). Evaluation metrics shown are cross validation (CV) and training accuracy (TA). The root relative squared error (RRSE) for each model is shown to elucidate how the model would perform using a simple predictor (where 0% is optimal). Gestural amplitude values, found within training data for static classifier models (using c.4000 examples per class), can be found in Table A3.

Gesture No.	Model	TA	RRSE TA (%)	CV	RRSE CV (%)	Class No.	No. Class Examples
1	k-NN	100	0.0117	100	0.0236	1	4018
	ABM1	100	0	100	0	2	4003
	DT	100	0	100	0		
	NB	100	0	100	0		
	SVM	100	0	100	0		
	DS	100	0	100	0		
2	k-NN	100	0.0011	100	0.0015	1	4042
	ABM1	100	0	100	0	2	4009
	DT	100	0	100	0		
	NB	100	0	100	0		
	SVM	100	0	100	0		
	DS	100	0	100	0		
3	k-NN	100	0.002	100	0.0036	1	3939
	ABM1	100	0	100	0	2	4033
	DT	100	0	100	0	3	3990
	NB	100	0	100	0	4	3997
	SVM	100	0	100	0	5	4083
	DS	40.5	86.5631	40.5	86.5631		

**Table 5:** Gesture 2 static classifier models and cross validation (CV) and training accuracy (TA) scores when post-processing is applied (across c.500–4000 examples per class), where percentage increase scores illustrate the improvement of CV/TA after post-processing (vs. before). Values in bold highlight the highest percentage increases of CV/TA scores when post-processing is applied and their attributed models.

Model	TA (%)	CV (%)	Examples per Class	% Increase in TA before Post-Processing	% Increase in CV before Post-Processing
KNN	100	100	500	0	0
ABM1	100	100		0.2	0.29
DT	100	100		0.2	0.29
**SVM**	100	100		**1.67**	**1.97**
NB	100	100		0	0
**DS**	100	100		**1.47**	**1.47**
KNN	100	100	4000	0	0.1
ABM1	100	100		0	0.01
DT	100	100		0.02	0.1
**SVM**	100	100		**1.85**	**1.89**
NB	100	100		0.39	0.39
**DS**	100	100		**3.27**	**3.27**

## Appendix A

**Linear Regression**: LR is a model that makes a prediction when observing the relationship between an independent (*x*) and dependent variable (*y*). It expresses the model prediction class as a linear combination of model attributes. It is especially useful when model attributes and class/outcome are numerical. It uses the least square method, derived from all model input data points, to find a line of best fit when modelling the relationship between the independent variable and dependent variable [55]. It is useful in interactive music practice because linear relationships between model input and output can create strict action–sound relationships; in this sense, they provide a reliable signal mapping when applying DSP to an audio signal. An LR approach can be expressed as follows, where $x_{1}$ represents the model input, $b_{0}$ represents the y-intercept, $b_{1}$ indicates the constant and *y* indicates the predicted value:
  (A1) $$\begin{array}{c} y=b_{0}+b_{1}x_{1} \end{array}$$
**Polynomial Regression**: A PR model is built upon the foundations of a linear regression model (finding a line of best fit linearly). However, a polynomial builds upon this by creating a line of best fit polynomially (using several terms to do so). This is done by increasing the exponent of the model input $x_{1}$. This model is useful in interactive music practice because it provides a less strict action-sound mapping (in comparison to the LR model). Due to the fact there are more terms, it gives the performer the option to use a ’smoother’ and non-linear way of mapping model inputs when applying PR model output to audio signals. A PR approach can be expressed as follows, where $x_{1}$ represents the model input, $b_{0}$ represents the y-intercept, $b_{1}$ and $b_{2}$ indicate the constant and *y* indicates the predicted value:
  (A2) $$\begin{array}{c} y=b_{0}+b_{1}x_{1}+b_{2}x_{1}^{2} \end{array}$$
**Neural Network**: The NN in Wekinator is an MPNN [56]. This model is based on a backpropogation framework to classify instances and is composed of a network of low-level processing elements, which work together to produce an accurate and complex output [57]. The NN model is composed of three core elements: an input layer (e.g., inputs from GIs in Wekinator), a hidden layer and an output layer. The backpropogation algorithm performs learning on a multilayer feed-forward NN [57]. At model training, it iteratively uses backpropogation to adjust the weights of the hidden layer(s), in order for the model to become more accurate. Model parameters, such as number of nodes per hidden layers, or number of hidden layers used, can be adjusted in Wekinator (see Table A8 for more information on NN parameters). An NN is useful in interactive music practice because the model gives a stabilised/smoothened model output value (in comparison to LR and PR models), due to the fact that the model uses a sigmoid activation function (mapping input values between 0 and 1) at the model output layer.

## Appendix B

**k-Nearest neighbour**: The k-NN model is a classification algorithm which makes predictions based on the proximity of new model input data to the assigned value of *k* [58]. If k=1, then the algorithm will look for the closest data point to the input feature (i.e., its nearest neighbour) and make a respective classification. However, if k=100, then the model will regard the 100 closest points to the input feature and make a respective classification; both approaches will disparately affect the classification outcome. Therefore, the model uses the value of *k* to assign a class to new data, based on the proximity of new data to the labelled data in the trained dataset.
**AdaBoost.M1**: The ABM1 algorithm is a popular boosting algorithm that takes weak classifiers/learners and improves their performance. This is done in Wekinator via choosing a base classifier: a DT or a DS (see Table A7 for more information). Initially, the ABM1 model assigns a weight to all features/attributes introduced to the model (all weights are defined by 1/n attributes). The weight is then iteratively redefined based on errors made on the training data by the base classifier chosen [59].
**Decision Tree (J48)**: The DT is a model based on the C4.5 algorithm used to generate DTs [60]. The DT graphically represents the classification process. Due to this, it is very useful for discovering the significance of certain attributes within datasets. In Wekinator, the DT is a univariate tree [61] that uses model attributes as tree internal nodes. It makes classifications by following a procedure: constructing the tree, calculating information gain and pruning. On construction, each model attribute is assessed in terms of its information gain and then pruned. Pruning a tree allows the user to discard any irrelevant information, within the dataset (attributes), when making classifications [61].
**Support Vector Machine**: The SVM model assesses observations, from model attributes, and their distance to a defined linear class margin; this is referred to as a maximum margin hyperplane [55]. This hyperplane aims to create a margin which achieves the best separation between classes and does not move closer to each class than it has to [55]. The observations that are closest to the margin are called support vectors. This classification method seeks to accommodate for low bias and high variance in the model. It also ensures that the model is not sensitive to outliers when new data are introduced to the (trained) model. However, the SVM model can also perform a non-linear classification (via the kernel chosen for the model, see Table A7 for all kernels available).
**Naive Bayes**: The NB algorithm is a classification model that ignores syntax and treats attribute observations as independent of the class [62]. It calculates the probability of observations belonging to a class by an attribute observation vector. This can be described as follows, where *P* denotes probability, $X_{i}$ is an attribute observation vector (at ith iteration) and *C* is a class label [62]:
  (A3) $$\begin{array}{c} P\left(X|C\right)=\Pi_{i=1}^{n}P\left(X_{i}|C\right) \end{array}$$
**Decision Stump**: A DS model is a single-layer DT that uses a single level to split an attribute from the root of the tree [63].

## Appendix C

**Table A1:** A table of post-processed model input values for static continuous models (using c.4000 examples per class), showing signal amplitude in relation to all performed gestures and respective model input types (i.e., pitch and EMG). All model inputs return a data range between 0 and 100. The minimum (min.) and maximum (max.) values for each parameter indicate gesture amplitude range. Boxplots showing the data distribution of each model input can be seen to the right of the table (model inputs are normalised within 0.0–1.0).

Gesture No.	Trained Model Input Parameter	Min. Value	Max. Value	Input Data Distribution
1	Euler pitch	49	72.92	
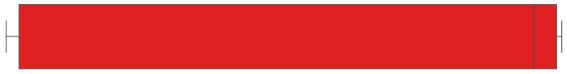
2	EMG1	2	6	
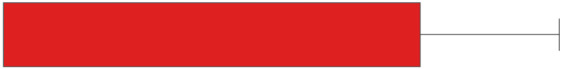
	EMG2	2	13	
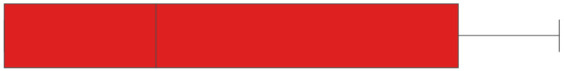
	EMG3	2	26	
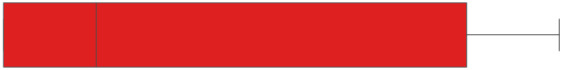
	EMG4	2	34	
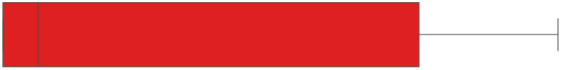
	EMG5	2	14	
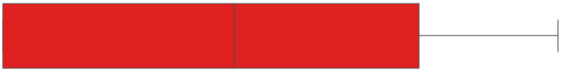
	EMG6	2	26	
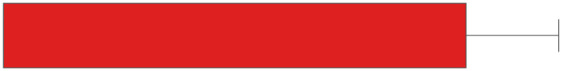
	EMG7	6	25	
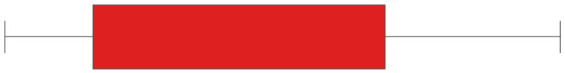
	EMG8	2	4	
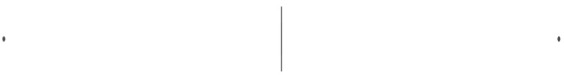
3 (i)	EMG1	2	5	
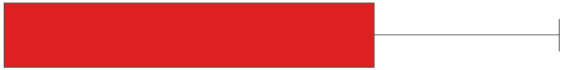
	EMG2	2	13	
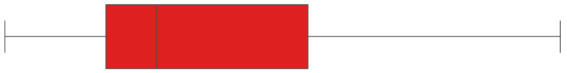
	EMG3	2	32	
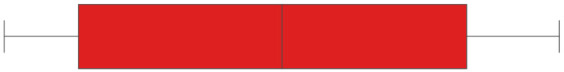
	EMG4	2	42	
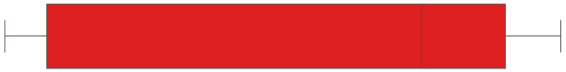
	EMG5	2	23	
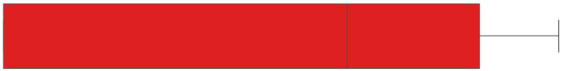
	EMG6	2	25	
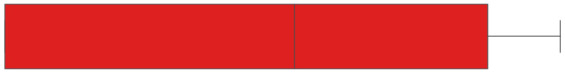
	EMG7	2	37	
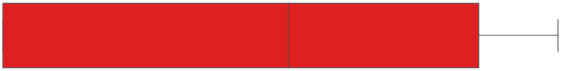
	EMG8	2	16	
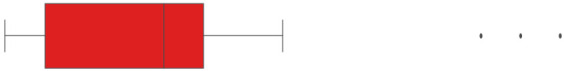
3 (ii)	EMG1	2	5	
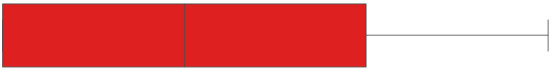
	EMG2	2	10	
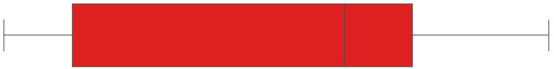
	EMG3	4	32	
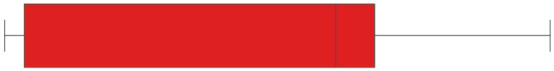
	EMG4	2	23	
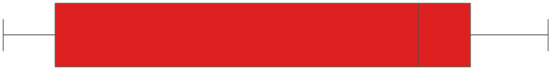
	EMG5	2	12	
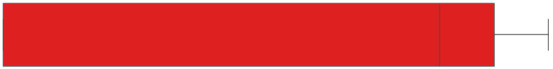
	EMG6	2	24	
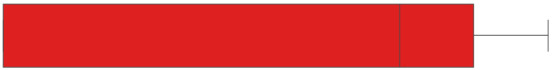
	EMG7	2	41	
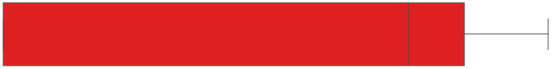
	EMG8	2	28	
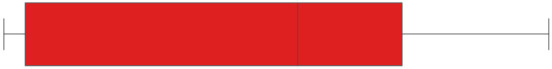
3 (iii)	EMG1	2	6	
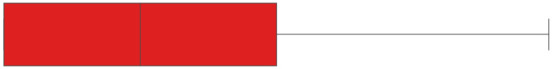
	EMG2	2	10	
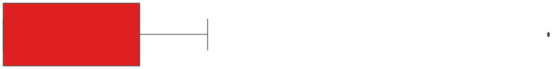
	EMG3	2	13	
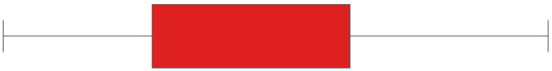
	EMG4	2	14	
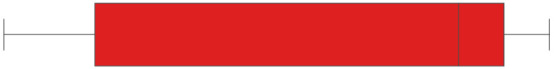
	EMG5	2	13	
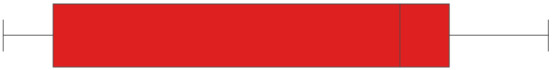
	EMG6	2	21	
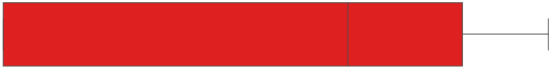
	EMG7	2	36	
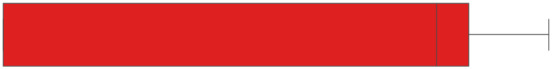
	EMG8	2	36	
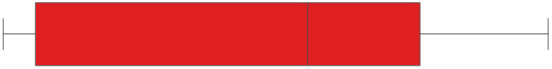
3 (iv)	EMG1	2	7	
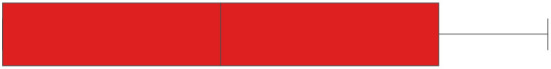
	EMG2	2	19	
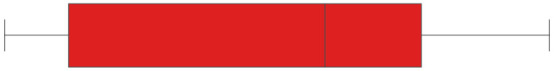
	EMG3	2	37	
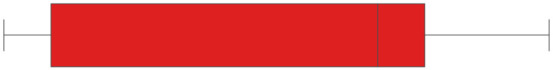
	EMG4	2	33	
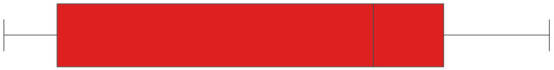
	EMG5	2	29	
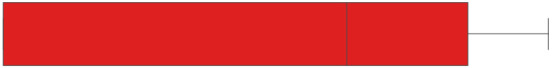
	EMG6	2	24	
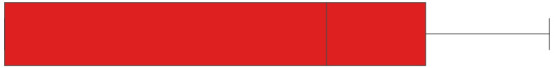
	EMG7	2	30	
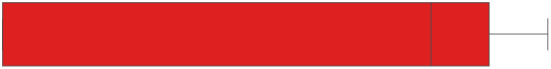
	EMG8	2	27	
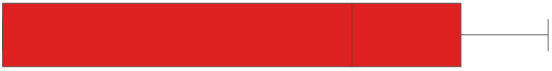
3 (v)	EMG1	2	6	
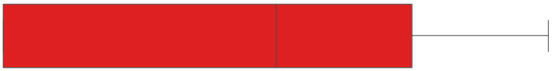
	EMG2	2	18	
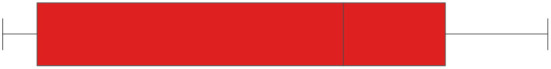
	EMG3	2	35	
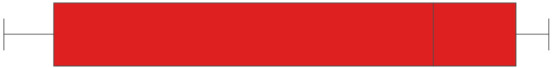
	EMG4	2	28	
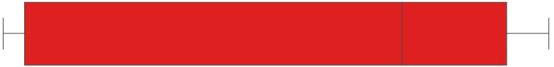
	EMG5	2	23	
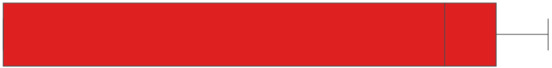
	EMG6	2	18	
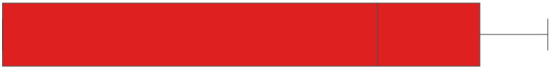
	EMG7	2	27	
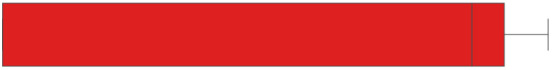
	EMG8	2	14	
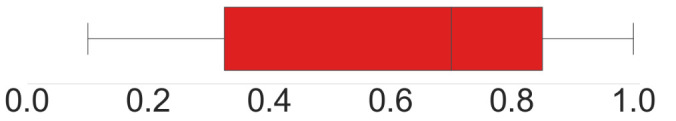

**Table A2:** A table of post-processed model input values for onset continuous models (using c.4000 examples per class), showing signal amplitude in relation to all performed gestures and respective model input types (i.e., pitch and EMG). All model inputs return a data range between 0 and 100. The minimum (min.) and maximum (max.) values for each parameter indicate gesture amplitude range. Boxplots showing the data distribution of each model input can be seen to the right of the table (model inputs are normalised within 0.0–1.0).

Gesture No.	Trained Model Input Parameter	Min. Value	Max. Value	Input Data Distribution
1	Euler pitch	48.88	72.05	
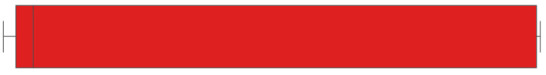
2	EMG1	2	6	
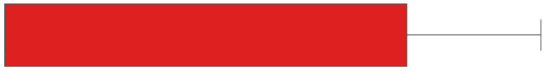
	EMG2	2	11	
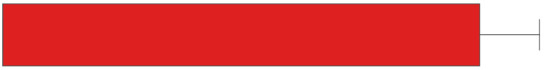
	EMG3	2	27	
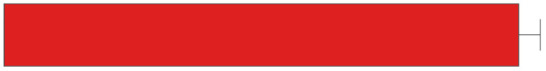
	EMG4	2	34	
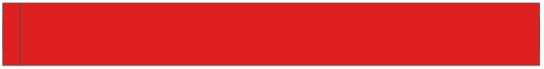
	EMG5	2	13	
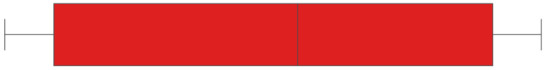
	EMG6	2	16	
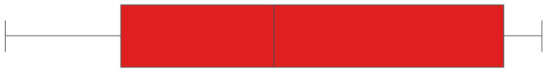
	EMG7	6	17	
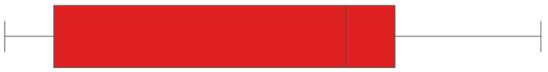
	EMG8	4	12	
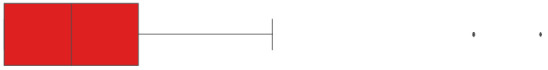
3 (i)	EMG1	1	8	
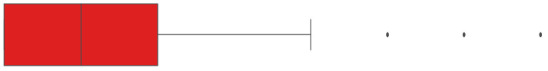
	EMG2	2	11	
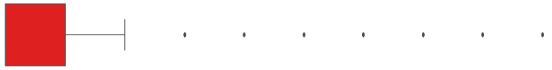
	EMG3	2	32	
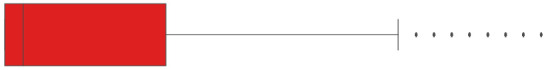
	EMG4	2	46	
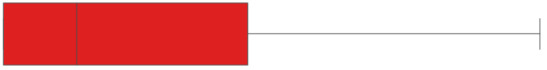
	EMG5	2	26	
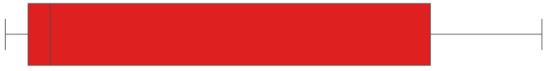
	EMG6	2	27	
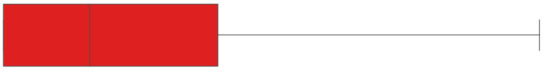
	EMG7	2	38	
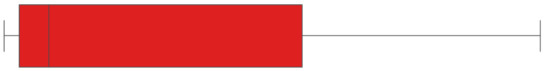
	EMG8	2	33	
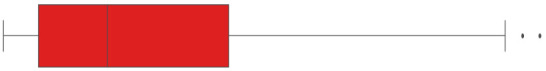
3 (ii)	EMG1	1	10	
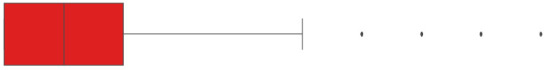
	EMG2	2	6	
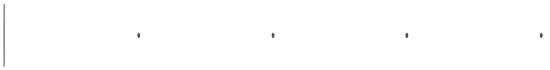
	EMG3	2	22	
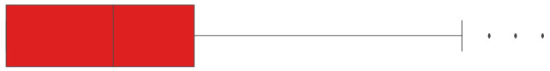
	EMG4	2	30	
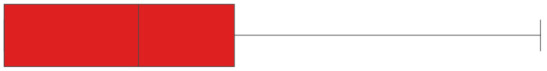
	EMG5	2	25	
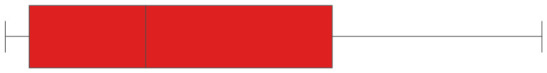
	EMG6	2	19	
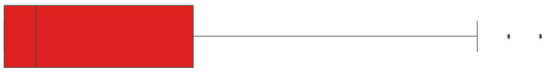
	EMG7	2	35	
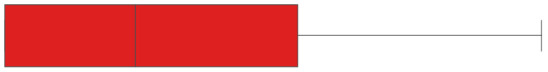
	EMG8	2	39	
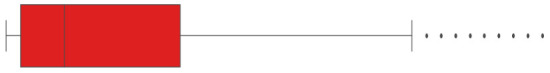
3 (iii)	EMG1	1	11	
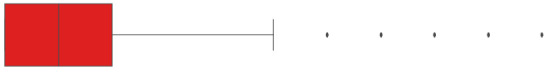
	EMG2	2	5	
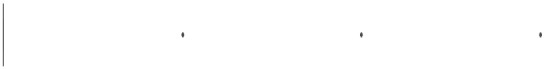
	EMG3	2	7	
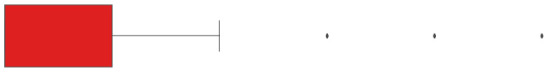
	EMG4	2	18	
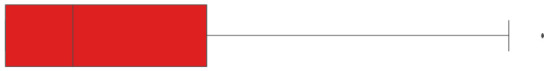
	EMG5	2	22	
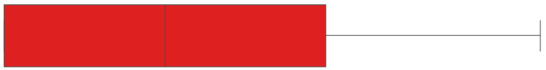
	EMG6	2	17	
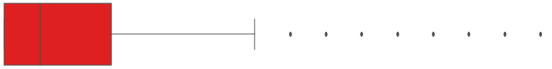
	EMG7	2	31	
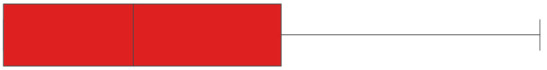
	EMG8	2	42	
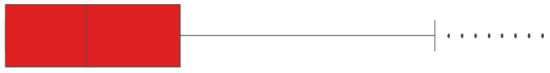
3 (iv)	EMG1	1	9	
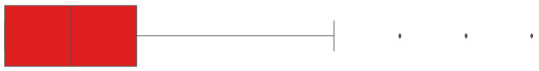
	EMG2	2	13	
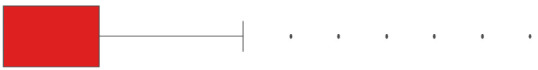
	EMG3	2	35	
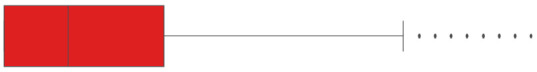
	EMG4	2	38	
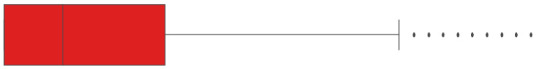
	EMG5	2	27	
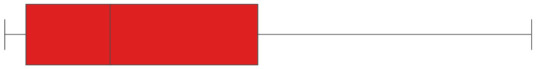
	EMG6	2	24	
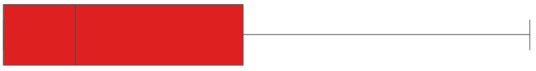
	EMG7	2	28	
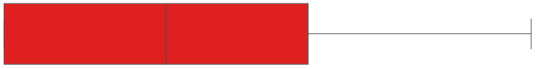
	EMG8	2	30	
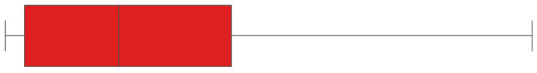
3 (v)	EMG1	1	7	
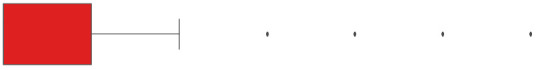
	EMG2	2	14	
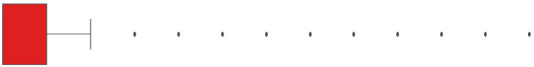
	EMG3	2	34	
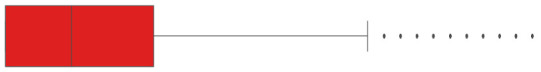
	EMG4	2	38	
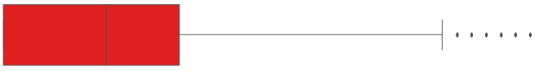
	EMG5	2	24	
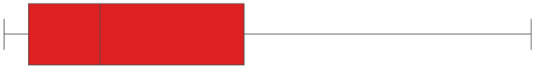
	EMG6	2	20	
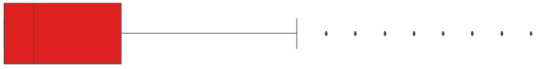
	EMG7	2	23	
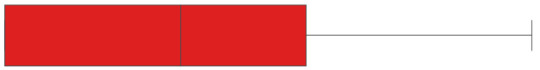
	EMG8	2	20	
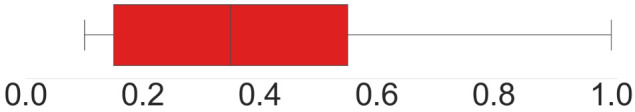

**Table A3:** A table of post-processed model input values for static classifier models (using c.4000 examples per class), showing signal amplitude in relation to all performed gestures and respective model input types (i.e., pitch and EMG). All model inputs return a data range between 0 and 100. The minimum (min.) and maximum (max.) values for each parameter indicate gesture amplitude range. Boxplots showing the data distribution of each model input can be seen to the right of the table (model inputs are normalised within 0.0–1.0).

Gesture No.	Trained Model Input Parameter	Min. Value	Max. Value	Input Data Distribution
1	Euler pitch	48.78	69.96	
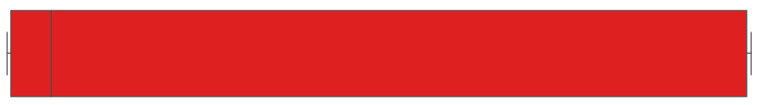
2	EMG1	2	6	
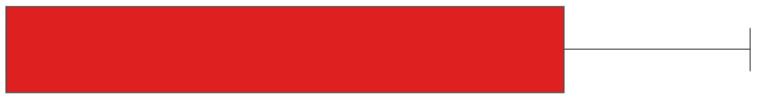
	EMG2	2	12	
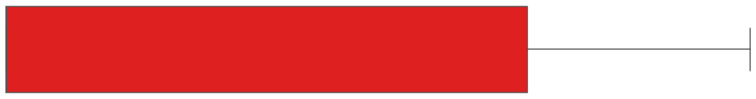
	EMG3	2	31	
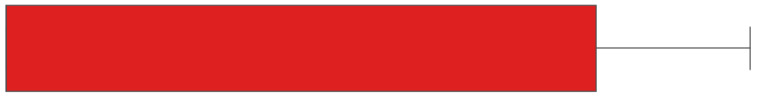
	EMG4	2	37	
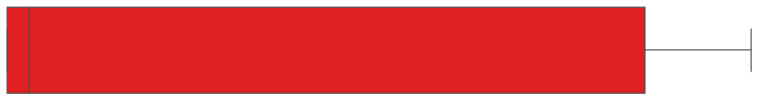
	EMG5	2	14	
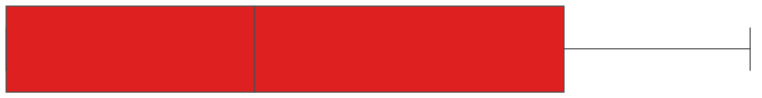
	EMG6	2	19	
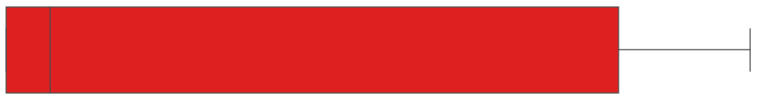
	EMG7	3	24	
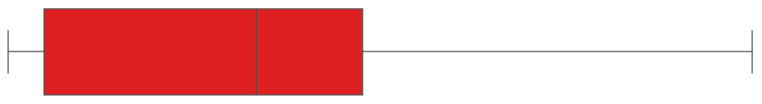
	EMG8	2	9	
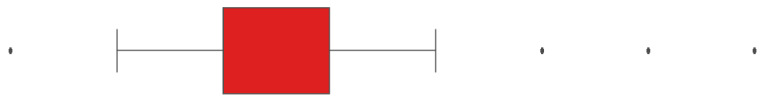
3	EMG1	2	10	
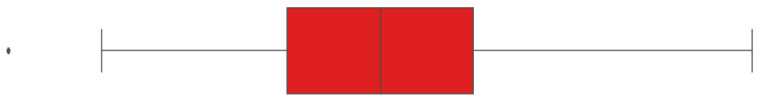
	EMG2	2	13	
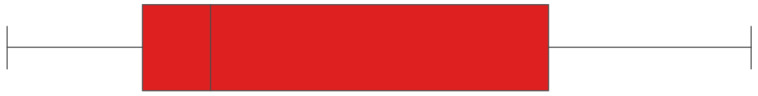
	EMG3	5	36	
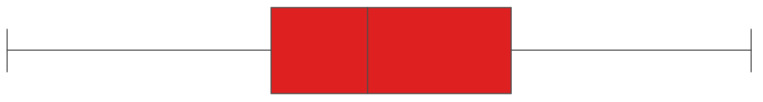
	EMG4	13	40	
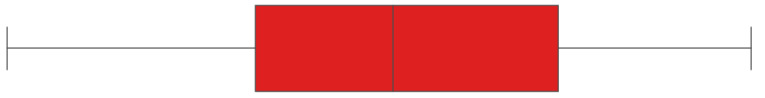
	EMG5	12	30	
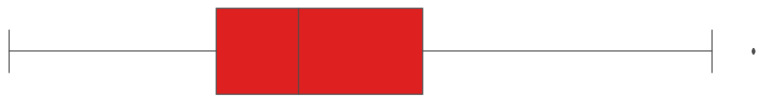
	EMG6	10	26	
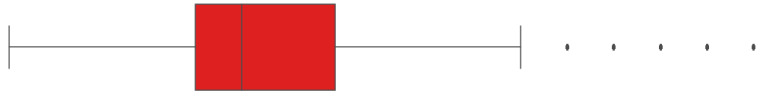
	EMG7	15	33	
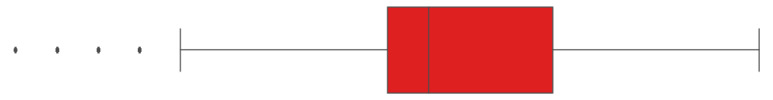
	EMG8	11	38	
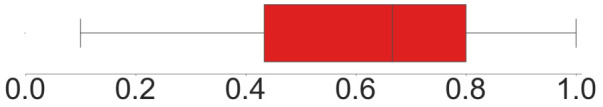

**Table A4:** A table of post-processed model input values for onset classifier models (using c.4000 examples per class), showing signal amplitude in relation to all performed gestures and respective model input types (i.e., pitch and EMG). All model inputs return a data range between 0 and 100. The minimum (min.) and maximum (max.) values for each parameter indicate gesture amplitude range. Boxplots showing the data distribution of each model input can be seen to the right of the table (model inputs are normalised within 0.0–1.0).

Gesture No.	Trained Model Input Parameter	Min. Value	Max. Value	Input Data Distribution
1	Euler pitch	47.92	70.81	
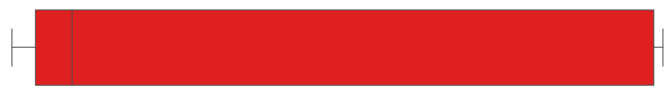
2	EMG1	2	6	
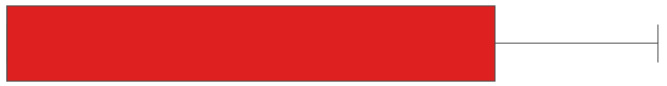
	EMG2	2	13	
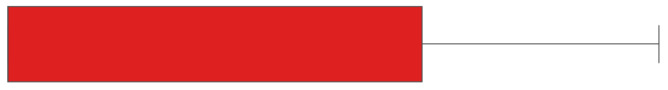
	EMG3	2	29	
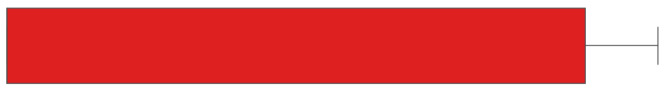
	EMG4	2	34	
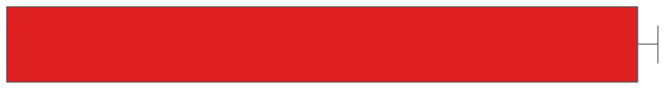
	EMG5	2	11	
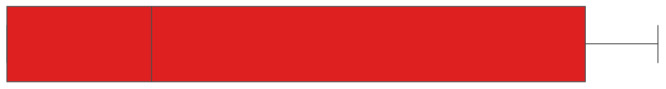
	EMG6	2	22	
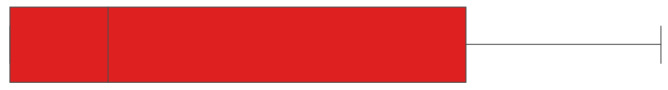
	EMG7	2	26	
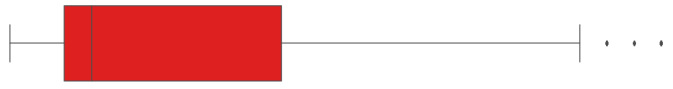
	EMG8	2	11	
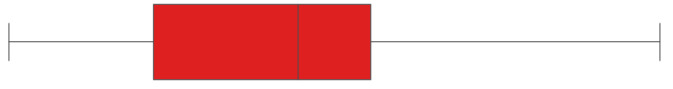
3	EMG1	2	14	
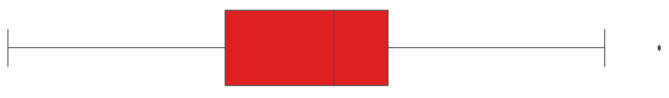
	EMG2	2	17	
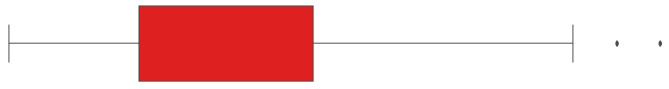
	EMG3	2	37	
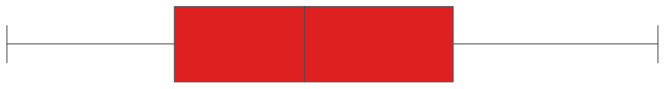
	EMG4	2	41	
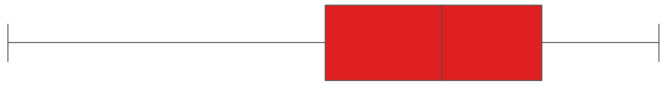
	EMG5	2	36	
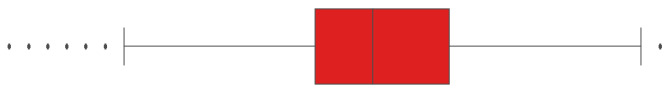
	EMG6	2	28	
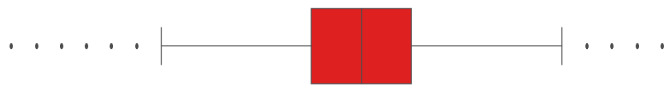
	EMG7	2	32	
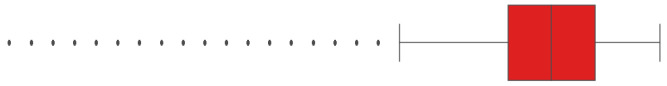
	EMG8	2	42	
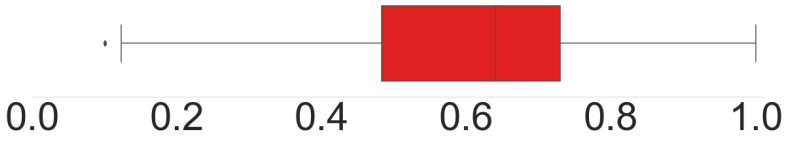

**Table A5:** A table of post-processed model input values for static DTW models (using c.300 samples per gesture over two examples), showing signal amplitude in relation to all performed gestures and respective model input types (i.e., pitch and EMG). All model inputs return a data range between 0 and 100. The minimum (min.) and maximum (max.) values for each parameter indicate gesture amplitude range. Boxplots showing the data distribution of each model input can be seen to the right of the table (model inputs are normalised within 0.0–1.0).

Gesture No.	Trained Model Input Parameter	Min. Value	Max. Value	Input Data Distribution
1	Euler pitch	69.83	70.20	
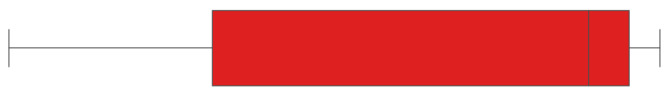
2	EMG1	5	6	
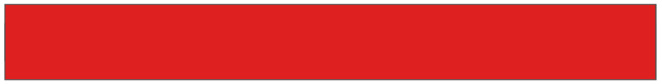
	EMG2	11	13	
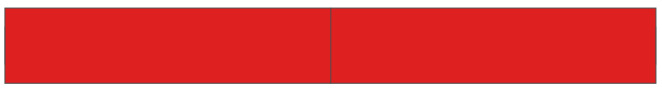
	EMG3	25	28	
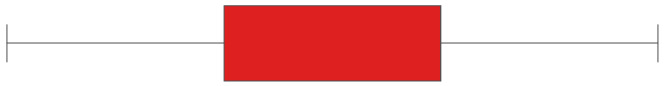
	EMG4	30	35	
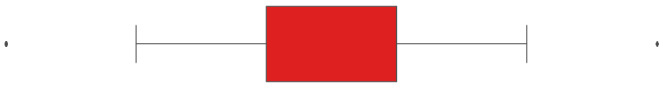
	EMG5	12	14	
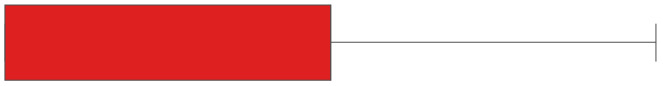
	EMG6	14	16	
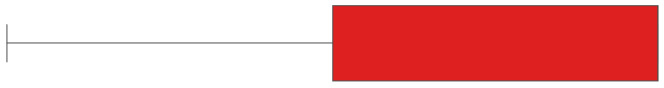
	EMG7	18	23	
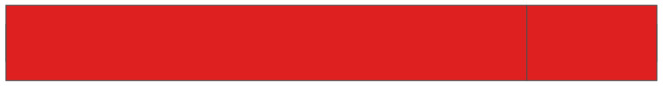
	EMG8	8	11	
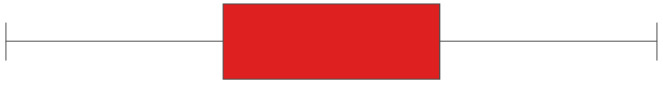
3	EMG1	2	7	
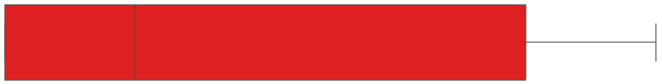
	EMG2	2	21	
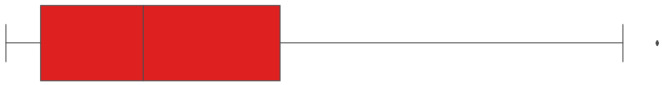
	EMG3	4	42	
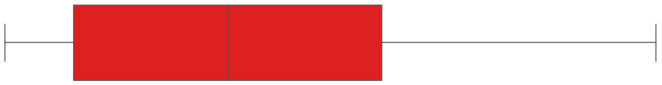
	EMG4	2	40	
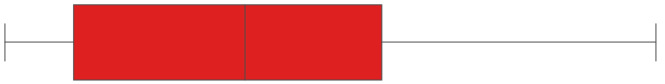
	EMG5	2	31	
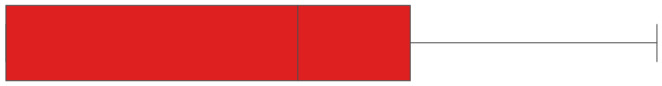
	EMG6	2	27	
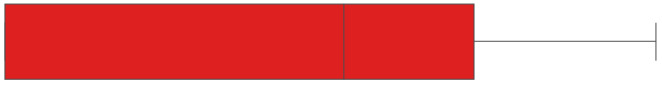
	EMG7	2	50	
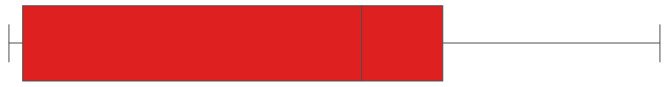
	EMG8	2	38	
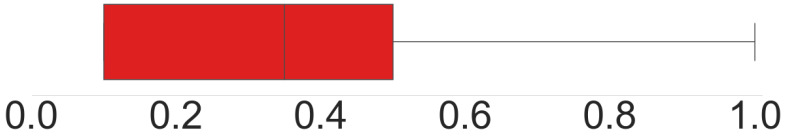

**Table A6:** A table of post-processed model input values for onset DTW models (using c.300 samples per gesture over two examples), showing signal amplitude in relation to all performed gestures and respective model input types (i.e., pitch and EMG). All model inputs return a data range between 0 and 100. The minimum (min.) and maximum (max.) values for each parameter indicate gesture amplitude range. Boxplots showing the data distribution of each model input can be seen to the right of the table (model inputs are normalised within 0.0–1.0).

Gesture No.	Trained Model Input Parameter	Min. Value	Max. Value	Input Data Distribution
1	Euler pitch	49.59	69.71	
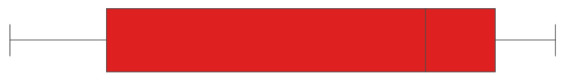
2	EMG1	2	8	
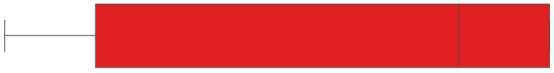
	EMG2	2	19	
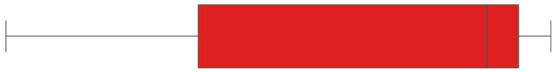
	EMG3	2	37	
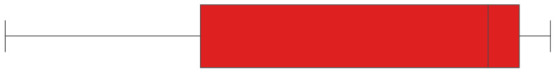
	EMG4	2	37	
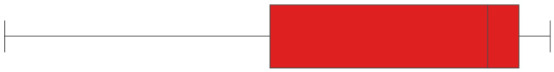
	EMG5	2	14	
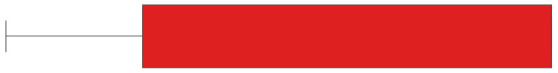
	EMG6	2	20	
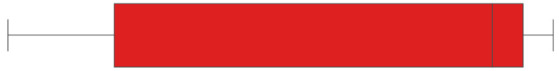
	EMG7	2	30	
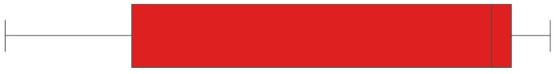
	EMG8	2	16	
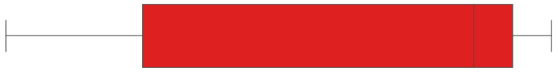
3	EMG1	2	12	
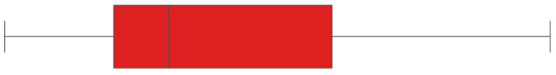
	EMG2	2	23	
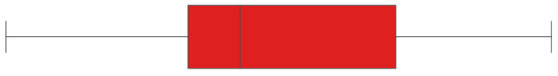
	EMG3	2	36	
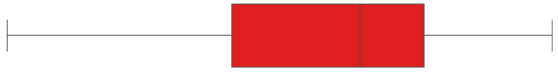
	EMG4	2	41	
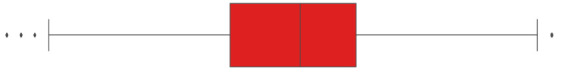
	EMG5	2	33	
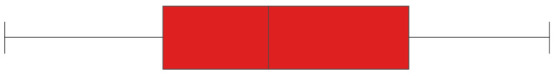
	EMG6	2	33	
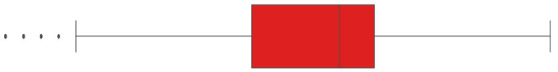
	EMG7	2	41	
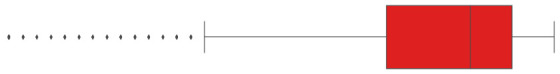
	EMG8	2	35	
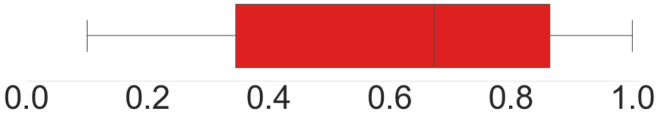

## Appendix E

**Table A7:** Available classifier models for model optimisation and their parameters in Wekinator.

Model Type	Model	Available Customisable Parameters for Each Classifier Model
Classifier	KNN	Number of neighbours (k).
	ABM1	2 base classifiers: decision tree and decision stump. Number of training rounds customisable for each base classifier.
	SVM	3 kernels to choose: linear, polynomial and radial basis function (RBF). Choice to use lower-order terms for polynomial kernel. Complexity constant modifiable for all kernels. Exponent of chosen complexity constant modifiable for polynomial kernel. Gamma of chosen complexity constant modifiable for RBF kernel.

**Table A8:** Available continuous models for model optimisation and their parameters in Wekinator.

Model Type	Model	Available Customisable Parameters for Each Continuous Model
Continuous	NN	Number of hidden layers customisable (maximum 3). Number of nodes per hidden layer, for number of hidden layers chosen, also customisable.
	LR	Three feature selection choices: no feature selection; M5 feature selection and greedy feature selection. The user can choose whether colinear inputs are removed—or not removed—for each feature selection.
	PR	Three feature selection choices: no feature selection, M5 feature selection and greedy feature selection. The user can choose whether colinear inputs are removed—or not removed—for each feature selection. The user can also choose the polynomial exponent (positive integer) number for each feature selection chosen.

**Table A9:** Available parameters for DTW model optimisation in Wekinator.

Model	Matches Computed:	Downsample:	Continuous Matches Use:	Other Parameters:
DTW	Continuously while running	So examples have max length of By constant rate Don’t downsample	A minimum length of	Match width
	Only once running stops		A min length equal to the shortest example	Match hop size
